# Factors related to university teaching that influence academic success of international medical students in China

**DOI:** 10.12688/f1000research.123281.1

**Published:** 2022-08-04

**Authors:** Qinxu Jiang, Hugo Horta, Mantak Yuen

**Affiliations:** 1 Xuzhou Medical University, Yunlong District, Xuzhou, China; 2 Social Contexts and Policies of Education, Faculty of Education, The University of Hong Kong, Pokfulam Road, Hong Kong SAR, China; 3 Center for Advancement in Inclusive and Special Education, Faculty of Education, The University of Hong Kong, Pokfulam Road, Hong Kong SAR, China

**Keywords:** academic success, international medical students, China, medical education, university teaching

## Abstract

**Background:** Academic success of international medical students enrolled in Chinese universities is of great significance, because it directly influences their performance in the license exam and in obtaining a job. Insufficient research has been conducted on academics’ awareness of factors related to teaching that affect their students’ academic success.

**Methods:** Semi-structured interviews were conducted with academics ( *N*=36) from November 2020 to January 2021 at two medical universities in China. Each interview, lasting between 30 to 70-min, was audiotaped, transcribed verbatim and analyzed using thematic analysis.

**Results:** The important teaching factors that academics perceived to influence the success of students are: (i) style of pedagogy, (ii) addressing students’ language difficulties and differences, (iii) teaching resources management, (iv) attributes of the academics, (v) supervision and guidance by the academic, (vi) rapport between the academic and student, (vii) linking teaching content to license exams, (viii) classroom discipline management, and (ix) assessment style.

**Conclusions:** University faculties and departments that are involved in teaching international medical students need to ensure that academic staff are provided with ongoing professional development and resources to enhance teaching quality. The nine areas identified above should provide priority topics for such staff training.

## Introduction

Over the past two decades, the internationalization of China’s universities ( Dang *et al.*, 2021; Jin & Horta, 2018) has seen medicine emerge as the most popular program after Chinese language for international students to pursue in Chinese universities ( Zhou, 2016). The Bachelor of Medicine and Bachelor of Medicine (MBBS) program taught in English medium is supported and supervised by China’s Ministry of Education (MOE) as well as being designed and implemented by universities ( Huang, 2014). An example of this is the release in 2020 of the document *Quality Control Standards of Basic Medical Education (in English Medium) for International Students in China.* This is from the China Education Association for International Exchange and the International Medical Education Committee, entrusted by the International Department of the MOE. The objectives of this standards document were to further standardize MBBS education across Chinese universities, effectively improve training quality and promoting a sustainable development of MBBS education.

45 universities are authorized by the MOE to recruit international students for medical education ( Xie, 2021). The World Health Organization (WHO) recognizes all these universities in the *Directory of World Medical Schools.* It means international graduates holding a Chinese MBBS degree are eligible to attend license exams in their home country. Examples include the Medical Council of India Screening Test, or the United States Medical Licensing Examination (USMLE), or the UK Professional and Linguistic Assessment Board (PLAB). This is perceived by students as one of the great advantages of studying MBBS in China—in addition to other perceived benefits that include less demanding admission requirements, affordable cost, and a safe, stable society ( Zhou, 2016).

Typically, the MBBS program takes six years to complete, with five years devoted to courses in natural science, Chinese language, basic science medicine, and clinic subjects, and one year of internship in Chinese hospitals or in students’ home country.

Most of the MBBS students originate from low-income countries from Asia and Africa, where the demand for health professionals is high ( Liu *et al.*, 2017). The majority of the students choose to return to their home country for career development after completing the MBBS program—except for some students who continue further studies ( Huang, 2019; Fan *et al.*, 2020). Jiang and Sun (2007) found that 87.4% of MBBS students planned to engage in medicine-related careers after graduation to make a significant addition to the health workforce in their home country, or they would migrate to other high-income countries ( Li & Sun, 2019; Han & Guo, 2009). The quality of MBBS education in China will likely influence their future license exam performance and the employment position they obtain. The reputation gained by these courses is the key to sustainable development of medical education for international students ( Zhou, 2016).

Medical license exams are fundamental to evaluate the learning of medical schools and their graduates ( Han & Guo, 2009). To some extent, the later success rate of MBBS graduates reflects whether China’s MBBS education meets international standards ( Mao *et al.*, 2012). In this respect, the results to date have been sub-standard when compared to those in several countries. For example, China has the most Indian students compared to other countries, who are required to pass the Foreign Medical Graduate Examination (FMGE) to practice medicine in India ( Muthyanolla, 2022). From 2015 to 2020, the total number of students sitting the FMGE from China was 43,632, but the average passing rate was only 12.51% ( Muthyanolla, 2022). Similarly, the passing rate of Nepalese students from China in Nepal Medical Council (NMC) Examination from 2016-2018 was only 34.3%, behind that of Nepalese graduates from Nepal (80.3%), Philippines (74.8%), Bangladesh (60.9%), Pakistan (58.4%), India (49.2%), Russia (44.6%), and Ukraine (40.9%) ( Aryal, 2018). These results indicate that the majority of China-educated MBBS graduates do not have the expected level of professional knowledge and skills.

Recent studies, while acknowledging the accomplishments of medical education for international students in China, have pointed out there are still several problems to be addressed. These problems are mainly identified as: lack of a unified training program for international medical students ( Liu *et al.*, 2021b), language barriers between lecturers and students ( Ding, 2016; Huang, 2019), ineffective teaching pedagogy ( Yang *et al.*, 2019a), insufficient well-qualified and experienced academics, and poor teaching resources ( Ding, 2016; Huang, 2019; Wang *et al.*, 2018), absence of robust quality assurance system ( Liang & Liu, 2019), classroom discipline issues ( Zeng & Sheng, 2019), and poor student quality ( Ma *et al.*, 2018; Huang, 2019). It should be noted that many of these studies were based on the authors’ own working experience and subjective reflection, so lacked empirical evidence. A few Chinese studies adopted a more objective quantitative method by surveying international *students’* opinions, but the views of academic staff were rarely solicited. The study reported here was designed to address this important issue by interviewing academics.

To the best of our knowledge, this is the first study in China delving into the beliefs of academic staff concerning pedagogical factors that influence success of international medical students. The findings may contribute to a better understanding of challenges faced by international medical students in China, and offer a direction for improving the quality of teaching in these universities. This is important for ensuring that graduates have the necessary expertise—and is very important for the reputation of the universities.

## Methods

### Ethics and consent

This study was approved by Ethics Committee of Xuzhou Medical University (XZMU20200028) and Institutional Review Board of Nanjing Medical University (NJMUIRB774) in [September] 2020. All participants gave written and oral informed consent to participate in the study.

### Research sites

The study was performed in two medical universities (A and B) located in Jiangsu Province, eastern China. Both universities had over 15 years’ experience in teaching international MBBS students, so it was reasonable to regard them as relevant sites from which to gather perspectives of academics engaged in teaching. A similar MBBS curriculum was adopted at these two universities, in line with the requirement by the MOE. MBBS students and Chinese students took separate courses and did not mix in the classroom. The courses for international students were taught principally through the English medium. The majority of their international students were from developing countries in Asia and Africa, and some students were far from proficient in English.

### Participants

Purposive sampling was adopted in this study. Criteria for selecting interviewees were: academic titles, years of teaching, gender, and course taught. The aim was to obtain a heterogeneous sample from the medical teaching body at the two universities. Academics were approached individually by phone call and WeChat invitation with the help of two “gatekeepers”. The target sample size before recruitment was around 30, but had to be increased until data saturation was reached ( Morse, 1991). A total of 40 academics were approached, but 2 of them declined the interview invitation and another 2 initially agreed but later withdrew by not responding to the researcher’s text messages.

In total, 36 academics (17 males, 19 females) were recruited, including 2 teaching Chinese, 3 teaching nature science, 11 teaching clinical subjects, and 20 basic medical science. The participants had between 2 to 13+ years of experience teaching international students; and all were responsible for teaching the same subject to both Chinese and international students.

### Data collection

Semi-structured interviews were conducted with each participant from November 2021 to January 2022. Only the first author and participants were present for each individual interview. Academics from University A participated in face-to-to-face interviews in their office or a small meeting room, where it was perceived to be comfortable and quiet, while online interviews were conducted with academics from University B (due to the coronavirus pandemic). Both face-to-face and online interviews were audio-recorded. Field notes were taken during the interviews. The interview questions, which were pilot-tested, addressed participants’ views on factors affecting student success, and their recommendations for improving teaching quality at classroom level and organizational level.

Before the interview, academics were debriefed concerning the purpose and significance of the study, and the likely length of the interview. Interviews were conducted in a quiet and comfortable environment. In conformity with ethical requirements, all interviewees were assured of anonymity and agreed to audio recording and dissemination of findings. Each interview, conducted in Chinese, lasted for around 30 to 70 minutes.

### Data analysis

A thematic approach was used to analyze data ( Braun & Clarke, 2006), with recorded audios later transcribed verbatim in Chinese. The Chinese transcripts were coded manually by the primary researcher, who immersed herself in the data and noted down her initial ideas on emergent themes. Initial codes were generated based on identified key words and phrases. Next, the codes were summarized into potential themes for each participant. The primary researcher translated the codes and themes into English. Then the researcher team discussed, adjusted and reached a consensus on final relevant categories. Trustworthiness was guaranteed by member checking, peer auditing and self-reflection. During the whole research process, the primary researcher kept a self-reflective journal to minimize the effect of her own background and work experience on the interpretation of the data.

## Results

### Theme 1: Teaching pedagogy

An overwhelming majority (86%) of academics perceived their teaching methods and practices to be a major influential factor on international students’ learning. They perceived a mismatch between their teaching approach and the learning characteristics of students. Most academics still relied on traditional teacher-centered lecturing, and tended to read verbatim from slides without properly explaining further or adding practical examples that could enhance the students’ comprehension of the subject. Some academics had noticed that lectures as a learning approach may not be suitable to international students’ learning needs. One academic teaching clinical medicine commented that in a lecture on clinical manifestations and diagnosis of diseases, the students were more concerned about the *treatment* and *recovery* from diseases. The academic suggested:

Students enjoyed learning about clinical *treatments* because they may think that it would be useful for their clinic work in the future. However, treatment is only briefly introduced in this class.

(Participant 25)

Unlike Chinese students, international students asked more questions and would interrupt the class to express opinions. This made the academics think more about how to communicate with them, and how to improve teaching quality. The majority of academics felt that much of the current teaching was spoon-feeding information and lacking interaction. Lecturers were failing to grasp students’ attention and hold their interest in class, which often affected their exam performance. Interviewees felt that teaching innovations such as case-based learning (CBL) and problem-based learning (PBL) should be used to teach international students, because this type of teaching mode was more heuristic, which would encourage students to learn more actively. They suggested CBL and PBL would increase the involvement of international students in learning activities. They could also make more frequent use of individual and group presentations and discussions, encourage asking questions, guiding students to think, and trying flipped class teaching (setting preparation assignments on a lecture topic *before* presenting the lecture). However, academics also felt that implementing these changes would be a challenge for them, because it would place more demands on their language ability, subject knowledge, ability to listen and respond to students, and control the class. It was pleasing to note that one of the few academics who already involved international students in interactive class learning activities reported positive changes in performance of these students:

International students are not interested in the traditional lecturing we use to teach Chinese students. Very few students listen carefully in class. I usually ask international students to summarize the content of the previous lesson in front of the whole class. They would become very active to participate and prepare well. I think international students like to show themselves in public. If I take advantage of this characteristic of theirs, I can stimulate their passion for learning.

(Participant 31)

Compared to Chinese students, international students were relatively weaker in basic knowledge and learning. Academics acknowledged the diverse academic abilities of international students and their different culture backgrounds and agreed that teaching approaches should be adjusted to these student characteristics. They also believed that it would be important for them to give culture-relevant examples in class that students were familiar with, make timely summaries of key knowledge points, give practice assignments after class, cover exam points, and provide timely feedback on assignments. An academic teaching radiology gave the example below:

For international students, you have to start from the basics. For example, in my [radiology] class, there is a CT value, high or low. High is bright, low is dark. You have to explain this foundation setting first; otherwise, they would become confused. Doing this sort of thing could help them, but would require academics to take more time to prepare for class.

(Participant 19)

Interviewees reported that real clinical cases needed to be integrated into basic medicine lectures, to help international students understand them better. They added that international students learn better with case examples. Academics without deep clinical experience but who are teaching biochemistry, microbiology, immunology, and parasitology found this to be a challenge. They suggested it was necessary for them to provide more than theory and facts by including relevant clinical knowledge to facilitate the learning of their students. An academic teaching biochemistry, with a background in biology, stated in this regard:

Students have asked me some clinical questions, which shows that they have this demand. This demand actually reminds me that I should include clinical knowledge in my teaching. This could make my class more attractive and make them realize that my course is very helpful to their clinical study in the future and can help solve clinical problems. In addition, it can make students realize the importance of my course, at least for now, and improve their learning enthusiasm. I’ve already started learning relevant clinical knowledge.

(Participant 6)

Academics also stated that their lecture slides probably need to be more interesting to attract students’ attention. The academics stated that there may be a need to use different teaching aids beyond writing on blackboard—such as videos, audios, and animations to make the classes more dynamic and understandable for students. An academic teaching pharmacology mentioned how he utilized online technology to make the learning in his class more effective.

I like to go to YouTube to find some videos about the mechanism of drugs to show in the class. Maybe my explanations are not very good, but the videos explain very well, which can help deepen students’ understanding. Moreover, I feel that playing videos when students are sleepy can increase their attention.

(Participant 22)

### Theme 2: Language barriers

After teaching pedagogy, the language barrier was identified as a significant challenge in improving the learning of international students. In discussion, 81% of the interviewees indicated that language differences and difficulties are negatively influencing teaching and learning quality. They also offered potential solutions. These Chinese academics spoke English as a foreign language, while international students spoke English as their first or second language (albeit far from perfectly). This is not a harsh criticism of either group, but merely highlights the actual situation. Mutual communication was constrained because the academics were not necessarily fluent in English, and the students were not necessarily fluent either. Academics found it difficult to understand students’ English accents and expressions, which may not be standard American or British English, particularly with students from India, Nepal, and Pakistan.

Academics revealed that while they could generally command medical English for in their own field after years of teaching, they still struggled with daily-life English and cross-disciplinary English. Limited English vocabulary outside their field affected their teaching, and was one of the main reasons why they often resorted to reading verbatim from slides rather than explaining concepts in their own words, and why they avoided engaging interactively with students. Consequently, students become bored during class, and sometimes were annoyed by poor communication. An academic teaching surgery compared teaching Chinese and international students concerning language in theory lectures.

If it is a Chinese class, the academic has many methods to teach. He has eloquence and rich experiences; he can solicit and quote from others when giving lectures. However, in case of teaching foreign students, due to the language barrier, academics cannot express themselves 100% smoothly. They can only communicate with students in the simplest way, so they cannot always respond appropriately to students. If they cannot attract students, students will lack interest in the class. Naturally, they do not devote themselves to learning.

(Participant 35)

Academics teaching clinical medicine who were also responsible for clerkship and internship at hospitals, indicated that it was hard to translate some Chinese words concerning a patient’s symptoms to English for the benefit of the students. Sometimes the translation made by an academic would not exactly match the symptoms. This situation was further exacerbated when international students could not comprehend explanations delivered in daily-life Chinese. Therefore, the effectiveness of clerkship and internship learning was adversely affected because of language barriers, as this academic teaching internal medicine explains:

Take the symptoms of angina pectoris for example. When I talk about angina pectoris to Chinese interns, I will use Xuzhou dialect, which is very easy to understand. However, it is difficult for me to convey the clinical symptoms reflected by most patients to foreign students through a more appropriate word. Because the pain reflected by the patient is not the oppressive feeling mentioned in the textbook. We cannot simply transfer the knowledge from books to students, but we have to combine it with our clinical practice. However, sometimes, it is very difficult to translate properly.

(Participant 25)

### Theme 3: Teaching resources management

Over half of the interviewees (58%) stated that issues with teaching resources also affected teaching and learning. Four sub-issues emerged as part of this theme.

***3.1 Demand of textbook and teaching materials***

Twelve academics complained that they lacked easy access to necessary textbooks, English question banks, and other relevant teaching materials such as reference books and videos. Universities did not mandate or provide any particular textbook for academics to use, and academics had to search for textbooks, or create their own materials by drawing on different English and Chinese sources. When the textbook of the academic was different from students’ own books, they could have difficulty understanding lectures. Sometimes, students were not assigned any textbook or relevant learning materials. Moreover, academics responsible for different modules or units in a course would often use different textbooks, which led to inconsistencies. Academics felt it is necessary for experts to develop good textbooks to help students understand and review lecture knowledge; and they suggested universities should purchase specialized English books, medical videos, and question banks for use by staff and students. One academic teaching gynecology commented on her difficulty with textbooks:

Our Clinical College once gave us some textbooks, but I think the versions of those books are relatively old. I have not seen updated books later. In those books, old methods of screening gynecological cervicitis, which were used one or two decades ago, were even introduced.

(Participant 27)

***3.2 Need for collaborative lesson planning***

Seven academics felt that it was important to engage in collective lesson planning for intellectual communication. This is lacking at present, but may be an important procedure for a teaching team to share experiences and discuss which knowledge points are the most important for students to master. In these planning sessions, application of flipped class teaching could be debated, and how to properly create synergies among different topics taught by different academics. Moreover, with collective lesson planning, new academics could be given a demonstration on how to teach international students by more experienced academics. Academics inexperienced in teaching international students expressed their needs to receive teaching tips on pedagogies, and have feedback from more experienced lecturers. However, in most departments, this collective lesson planning and guidance was lacking, particularly clinical medicine subjects because the teachers were busy with their own clinical and research work:

Theoretically, there should be collective lesson planning, but it cannot be done in practice … Teachers have their own understandings of knowledge and lecturing methods, and collective lesson preparation has little impact on them … Basically, teaching has been weakened and become a mere formality. There is no teaching demonstration for new teachers any more. The supervision mechanism has gradually disappeared. Now the overall orientation no longer takes teaching as the main evaluation index, but mainly takes SCI publications.

(Participant 24)

***3.3 Weak teaching supervision system***

Seven academics reported that universities lacked a sound supervision system for teaching quality, so academics might well conclude that it makes no difference if one teaches well or not. Without a system that has rewards for good performance and penalties for poor performance, academics would have no incentive to teach well. Strengthening supervision of teaching by including regular observation by experts and spot checking of lecture quality was something that some academics desired.

The School of International Education needs to establish a system or assessment standard, and implement rewards and punishments in each teaching and research department. If there is no difference between good and bad teaching, teachers will not be motivated.

(Participant 33)

***3.4 Inadequate clinical teaching resources***

Five senior clinical academics mentioned there was a shortage of well-qualified persons to teach theory, clerkship and mentoring internships in hospitals. In some departments, due to high turnover of staff, there was lack of a stable teaching team responsible for international students. Recently hired academics and new doctors who had minimal or no prior teaching experience were asked to teach these students. Their teaching would be limited to reading their notes or slides aloud, without explanation or discussion. Academics with teaching experience hoped universities and hospitals would attach more importance to consistency in teaching international students, and to introduce incentives to encourage young, qualified doctors to participate in teaching. There was a perceived need to train new academics for future teaching work:

During the clerkship, we had two teachers to teach 25 international students. The venue space and teachers were not enough … Internship was more like clerkship learning just for a visit in the case of foreign interns, because some teachers could not speak English and they were very busy. Foreign interns could not operate due to doctor-patient relationship concern. They could not receive much guidance … You know, this is a problem.

(Participant 24)

Three academics also mentioned that their course syllabus was obsolete. A senior academic who teaches pediatrics said it was imperative to discuss with international students to know which diseases they needed to learn about for work in their own country. The course syllabus could then be updated accordingly. This would make it easier for students to relate to the content being taught in class. In this regard, academics suggested their departments should evaluate their existing syllabus, and improve it where necessary by making it more relevant.

### Theme 4: Attributes of academics

Approximately 50% of interviewees disclosed that the attributes of academics, such as their dedication, passion for teaching, attitude, sense of responsibility, and subject knowledge played a pivotal role in ensuring teaching quality that helps students learn. The academics interviewed commented that much more time and effort is needed to prepare lessons for international students because of the modifications required. Some perceived the workload of preparing a lesson in English was three to ten times greater than preparing a lesson for Chinese students. For instance, they would have to write a lesson script beforehand to read in aloud in class, because it was too challenging to improvise their English during the session. This type of language barrier would not occur in the Chinese class.

The interviewees admitted that many academics were not motivated to engage in teaching international students because universities *prioritized research* over teaching. There were no incentives for academics to devote time and effort to areas that would not advance their careers. Therefore, they felt more inspired to do research and were much less driven to teach international students, even though they were qualified. Moreover, some academics might become apathetic about teaching, which would affect the quality of their contribution. A typical observation was:

All materials for lesson preparation must be in English. It is already very difficult to prepare lessons in Chinese. I have to prepare lessons in English and practice my manuscript … In this current university environment, which generally attaches importance to scientific research rather than teaching, everyone is willing to focus on their own research rather than pay attention to teaching. Research awards are more tempting… When it comes to professional rank evaluation, papers are more useful than a teaching competition award.

(Participant 3)

Despite the common perception of heavy workload, challenges, and dissatisfaction with a lack of incentives, some academics maintained a personal commitment to their teaching out of a sense of responsibility and self-improvement.

To maintain the enthusiasm of academics in teaching, it is not about monetary stimulation, but a sense of value and honor. Academics are not unsentimental people … Many academics are dissatisfied, but they continue to teach even if they are not satisfied. This is dedication, awareness and sentiment … I don’t want to talk about interests, but the school should give me some other aspects that emotionally make me feel honored to teach international students.

(Participant 4)

When asked about what incentives universities could provide to motivate academics, the interviewees claimed that instead of placing MBBS education in a peripheral position, university management should attach more importance to MBBS education and to the academics who provide it. They expressed strong need for continuous professional learning to improve pedagogies, and assistance with oral English through formal or informal learning opportunities at home or abroad. Several academics claimed that a university should count their teaching performance as part of any evaluation for promotion, rather than solely basing it on number of publications. This would ensure that academics would become more motivated and enthusiastic to teach. A few academics also desired funding opportunities to implement teaching innovations in the MBBS program, but it was not possible. Two academics hoped the university could count their class hours as double or three times more than Chinese class hours, because they spent much more time on lesson preparation and had a need of class hours for academic promotion.

### Theme 5: Teacher supervision and guidance

When asked what academics could do to help students improve their academic performance, nearly 50% of the interviewees indicated that there was a need for them monitor their students’ progress more closely, and to push underperforming students harder to be responsible for their study. Students could be lazy and lack self-discipline. Academics felt that if they could devote more time into supervising low-achieving students and talking with them after class to encourage them, the students may feel they are being cared about and be more motivated to study. An academic teaching cell biology highlighted the importance of encouraging and caring about students:

Students are still children. If academics communicate with them more, they will feel pressure and study under pressure … In the first year of my teaching, I dedicated all my energy to push them. I tried my best to memorize all the students' names and faces. They felt my care. Several students still have been in contact with me since graduation … I was busy with my own life and family, but if I had spent more time at that time, I could have saved a few more students from failing.

(Participant 21)

Moreover, six academics felt it was not only important to impart knowledge and skills to students, but also moral and ethical education, values and responsibility in class. They stated that it was critical to educate students to cherish learning opportunities in China, correct their learning attitude, and be responsible for medicine. By doing so, students’ intrinsic motivation could be stimulated with a sense of respect and responsibility, as stated by an academic teaching anatomy:

In anatomy class, the specimens are donated by others. No matter the country in the world, the donors are the worthiest of respect. As a medical student, you should respect them and patients, because they, ordinary people, have given us the opportunity to learn and explore medicine. You should treat patients well in the future. Why did they donate their bodies? Because they hoped you could become a good doctor.

(Participant 28)

### Theme 6: Teacher-student rapport

Approximately 31% of the interviewees mentioned that getting to know about students and their cultures and establishing good teacher-student rapport would be conducive to better communication and teaching. In particular, communication between academics and students could help build emotional connections and understanding in class. For example, without knowledge of students’ lives and cultures, any academic may accidentally say culturally insensitive statements that can cause discomfort. An academic teaching orthopedics with more than ten years’ teaching experience with international students shared how he successfully developed a good relationship with students by incorporating students’ cultures into class.

Once, as soon as I finished watching the Indian film *Dangal*, I quoted 10 classic sentences into my slides to motivate my students in class. They became very interested when they saw these quotes. “Wow, this is our Indian movie.” Sometimes, I talk about my feelings about Nepalese festivals in class. They feel excited and think the teacher acknowledges their culture… If students like you, they will learn your course well.

(Participant 18)

An academic teaching anesthesiology mentioned that academics teaching clinical subjects generally knew less about their students, and thus they failed to establish good rapport. This made their lectures seem rigid and lacking in attention to the students as individuals. The academics teaching clinical medicine suggested that universities should arrange activities to increase communication between staff and students. This is particularly important for younger academics.

### Theme 7: Detachment of teaching content from license exams

Over a quarter (28%) of the interviewees pointed out that one of the primary reasons why many international students lack interest in learning is probably because the current course content and resources do not correspond closely enough to the demands made in the license exams. Academics usually teach the knowledge that they think is important, and this may not precisely match a curriculum based on the license exam. What is required is a blending of both academics’ specialist knowledge and a prescribed curriculum. This issue was critical, because students would be more motivated to learn things if they perceived them to be useful to them in passing examinations, and in their working life. However, some academics argued that it would be difficult to address license exam needs of students from diverse countries, where exam content may vary. In this situation, it seemed essential to develop a more contextualized curriculum, as explained by an academic teaching anatomy:

One of the key points of our teaching is to help students pass license examination. If a student does not pass the exam, he/she will not even qualify for matching hospitals. This is true of both Chinese students and foreign students. Teaching and license examination cannot be separated. If we want to help students pass the license exam, teaching activities should play a guiding role … The passing rate of license exam is the most important measure standard of a medical university. Practice is the sole criterion for testing truth.

(Participant 2)

### Theme 8: Classroom discipline management

Chinese academics felt frustrated and perplexed by international students’ certain behaviors in class. International students were often late for class, left class in the middle for no reasons, listened to music, used smartphones, or would chat with others and made noise. These behaviors negatively influenced the mood and positivity of academics towards international students, as by comparison such discipline problems would rarely occur in Chinese students’ class. Seven academics (19%) stated that it might be students’ own cultural habits, but they should follow Chinese rules. These academics dedicated certain time to disciplining students in class to make students more serious about study. However, more academics emphasized that it was the student administrators’ responsibility to strengthen student discipline management. One academic teaching hygiene explained how she managed class discipline to push students learn.

In sharp contrast to Chinese students, foreign students pay too little attention to class discipline… For example, for morning class at 8 a.m., some students cannot come until 9 a.m. I wondered why. Later, I went to Pakistan for a visit. When I went to a university, I understood that this was their habit. However, in China, if you want to obtain a Chinese degree, you have to follow Chinese rules … I count the check-in and lateness into class participation score, and I will call the roll five minutes in advance. Sometimes I close the back door in class for fear that they might sneak out. Sometimes I put roll call on the second quarter of class to prevent someone from sneaking out after signing in … It is like fighting a battle of wits and courage with them.

(Participant 30)

### Theme 9: Assessment styles

Six academics (17%) stated that the current assessment system was problematic, mentioning the exam evaluation system and the final elimination system.

***9.1 Exam evaluation system***

There is no formative assessment but only a summative assessment based on final exam performance. Academics suggested that the university add formative assessments such as a mid-term exam or in-class quizzes, because it could help academics to follow students’ progress and difficulties, and push students to study harder, and decrease the final exam pressure on students.

One academic teaching pathophysiology mentioned that the evaluation of international students’ academic performance should take into account of their characteristics rather strictly follow the same evaluation system of Chinese students. This academic felt that international students were more interested in practical learning in the laboratory and generally outperformed Chinese students, so the university could consider increasing the weight of practical score into their final course score.

Academics also mentioned they wanted to change the assessment methods, but in most cases, it was beyond their control unless the changes were approved at the organizational level after going through complicated application procedures. One academic teaching parasitology exemplified how their department improved their assessment method based on students’ feedback.

Students came to complain that the exam was unfair because some students did not attend class, just memorized key points the week before the exam and got higher marks. After receiving their feedback, we improved. We took into their formative performance as a part, and the final exam accounted for only 50%. Answering questions and signing in to class are all part of the formative performance. I think we should add formative assessment and tell students to listen to the whole class. Our purpose is not to let them fail in the exam, but to guide them to learn something. Students understand this. Finally, almost all students passed my course.

(Participant 29)

***9.2 Final elimination system***

Seven academics pointed out university assessment and evaluation systems were less strict with international students. It was perceived as a loose entry and exit to the course, which would have no pressure on students to study. These academics suggested the university should implement a final elimination system to delay graduation or fail those who are disqualified for graduation and a MBBS degree.

The university must strictly enforce the examination system and cannot deliberately make exceptions in international students’ favor. Schools should dare to weed out unqualified students, so that they will be afraid. Once they are afraid, most of them will study hard.

(Participant 10)

## Discussion

From the standpoint of academics, this study revealed critical factors that are worthy of attention with respect to academic success of international students in China. Nine key themes emerged covering challenges that include teaching pedagogy, English language, teaching resources management, teacher attributes, teacher supervision and guidance, teacher-student rapport, detachment of teaching content from license exams, class discipline management, and assessment styles.

It is evident that teaching methods and language barrier were the most frequently reported factors that impacted Chinese academics’ teaching effectiveness and international medical students’ academic success. This finding is in keeping with that reported in the systematic review by Dang *et al.,* (2021), in that these are the most common challenges facing Chinese academics in English-medium-instruction (EMI) programs. The finding of teaching method as the most dominant factor perceived relevant to student academic success, is aligned with the findings of Helm and Guarda (2015). They reported that in an Italian university, most lecturers in programs where English is the medium instruction needed to improve their teaching competences. The literature also indicates that the pedagogical abilities of academics seem to be greatly influenced by language barriers ( Ding, 2016; Huang, 2019; Zhan, 2017). Yang *et al.* (2019b) found EMI teachers in a Chinese university, more than non-EMI teachers, became more didactic and less interactive in class, which then hindered teaching and learning. Despite this, improved English skill does not necessarily equate to improved pedagogical changes ( Dafouz, 2018; Dang *et al.*, 2021; Helm & Guarda, 2015). The finding of this study suggests that while it is important for the university to address the oral English abilities of academics in both English related to a specific discipline and English in daily life, it is more imperative to focus on improving the pedagogy of these academics as key to academic success of international medical students. Universities should offer ongoing professional learning in teaching approaches such as PBL and CBL for pre-service and in-service academics, taking full consideration of the needs, ability and interests of international medical students ( Liu *et al.*, 2021b; Saleh *et al.*, 2012; Yang *et al.*, 2019a). Meanwhile, universities should strongly encourage and support teaching innovations with sufficient funding.

This study uncovers that limited access to teaching resources largely inhibits teaching and learning. The lack of uniform English textbooks and relevant teaching materials for international students has also been identified in many universities in China ( Huang; 2019; Jiang *et al.*, 2014; Yang *et al.*, 2019a; Zeng & Sheng, 2019), and in other developing countries, such as in a Ukrainian university where EMI programs were taught to domestic students ( Goodman, 2014). It is acknowledged that textbooks are still the main carrier of subject content, therefore, the selection and use of textbooks will directly affect the teaching and students’ knowledge attainment ( Li & Cui, 2010; Li *et al.* 2013). Compared with the rich curriculum integrated teaching resources in developed countries in Europe and the United States, China still lacks a set of English medical teaching materials with complete independent intellectual property rights and in line with the characteristics of its medical education in terms of pertinence and adaptability ( Wang *et al.*, 2018). The dire shortage of English teaching resources has limited the sustainable development of medical education for international students in China ( Wang *et al.*, 2016). Although some Chinese universities have started to write teaching textbooks on their own, and have made progress, there are also some problems. For example, the English level of Chinese teachers is uneven, and the writing style cannot be unified ( Wang *et al.*, 2018).

In alignment with existing literature ( Ding, 2016; Huang 2019; Yang *et al.*, 2019a; Zeng & Sheng, 2019), the findings of this study show that lack of collaborative lesson planning for MBBS classes, shortage of clinical teaching resources, and weakened supervision mechanisms are prevailing in Chinese universities. With regard to the issue of perceived obsolete syllabus, Zhou (2016) shows that Tianjin Medical University has added relevant teaching content according to changes of the disease spectrum in the country of origin. For instance, contents such as *Schistosoma aegypti* and *Schistosoma mansoni* which are prevalent in Africa were added to the course of Human Parasites. The case of Tianjin Medical University implies that organizational support plays a critical role in guaranteeing the quality of MBBS education.

Blömeke *et al.* (2016) found the quality of academics in terms of their experience, education background, motivations, professional knowledge and self-efficacy to predict student achievement. Similarly, the finding of this study suggests that the attributes of academics such as passion for teaching, professional responsibility, dedication, attitude, and content knowledge are perceived to have an effect on teaching quality. As indicated by the interviewees, academics are often demotivated to teach international students because of a lack of emotional, material, and career incentives. Lacking incentives seems a prevalent phenomenon, as reported by other universities in different provinces in China—to name a few, Liaoning Medical College ( Ding, 2016), Three Gorges University ( Zeng & Sheng, 2019), and Zhengzhou University ( Zhang, 2016). This problem was also found in a study in Sweden ( Stenfors-Hayes, 2010). This suggests that university policy makers need to be attentive to the opinions and needs of academics if they aim to formulate an appropriate reward system that can promote teaching quality for student success.

The finding concerning supervision and guidance for students and its effect on student success is new to the literature. Chinese academics subconsciously assumed a parental role to discipline international students, which implies their commitment to education. Moreover, moral and ethical education for international students during and outside lectures has been recognized in Chinese studies ( Liu *et al.*, 2021a; Wang & Huang, 2019). This may be more relevant to a Chinese context because of the influence of Confucian ideology in Chinese education that academics should not only impart knowledge and skills but also bear an obligation to cultivate the mind ( Ye, 2001).

Similar to the findings of previous studies in China ( Yang & Lei, 2021; Zhao, 2021) and Iraq ( Saleh *et al.*, 2012), teacher-student rapport is an overlooked dimension in MBBS teaching. Lack of good teacher-student rapport, due to differences in language, cultural background, and religious beliefs, could make the dynamics within a classroom dull and rigid ( Yang & Lei, 2021). Building a good relationship between academics and their students could help foster a classroom environment that is favorable to learning. This requires academics to learn more about the life and learning characteristics of international students, and to communicate with them more after class to establish a harmonious and supportive teacher-student relationship.

The issue of teaching course content that is detached from license exam requirements has been recognized in some Chinese studies ( Han & Guo, 2009; Huang, 2019). The universities with more capabilities have taken action to integrate license exam content in their teaching. For example, the MBBS curriculum at Zhejiang University is internationalized by reference to UK medical education standards and clinical executive standards in *Tomorrow’s Doctors* ( Fan *et al.*, 2020), United States Medical Licensing Examination, and Professional and Linguistic Assessment Board. Li and Cui (2010) from Dalian Medical University suggest introducing license exam tutoring by inviting foreign academics to teach Chinese academics by studying exam questions and content; but this was seen as separate from regular teaching. This difference between the two cases implies that university staff should certainly prepare students for license exams, but also shape the curriculum to include topics in curricula tested in license exams (e.g. FMGE).

Classroom disciplinary issues with international students are common and can negatively influence the lecturing style of academics. This was also identified in other studies ( Jin *et al.*, 2009; Zeng & Sheng, 2019). Some academics take disciplinary action with problem students while others ignore the behavior but feel uncomfortable with the lack of control. In a survey of learning experience in a medical university in Southwest China, half the MBBS students felt academics were not strict enough with class discipline ( Yang & Lei, 2021), probably because of language barriers. Yet, the finding of this study suggests that academics should try to ensure good disciplinary management for the sake of well-behaved students. Action needs to be taken with misbehaving students to focus their attention on attendance and listening during lectures.

The finding on the effect of assessment style on international medical students’ academic achievement has scarcely been discussed in the literature. In one study conducted in Ningxia Medical University, Zhang *et al.* (2020) focus on how the implementation of formative assessment in their MBBS microbiology course helped improve class attendance and learning attitude, develop creativity and self-directed learning habits, increase teacher-student communication, and make evaluation fairer and more objective. This case shows the advantages of revising assessment styles. Notably, Zhao *et al.* (2020) claim that the current assessment of students in medical colleges and universities in China is generally based on summative assessment (at the end of the course), and that there is a lack of understanding of and attention to formative (ongoing) assessment and evaluation during the course. Summative assessment is seen by the interviewed academics as a relatively single and weak assessment method that could reduce students’ enthusiasm for learning. Assessment only at the end of a course makes it difficult to monitor progress in students’ growth in professional knowledge ( Zhan, 2017). Therefore, it is important for universities to transform assessment styles appropriately to help international medical students learn.

In congruence with other studies ( Wang, 2013; Zhang & Hu, 2019), it was noted that there was often a tendency in Chinese universities to graduate international students by lowering exam difficulty or pass mark, or modifying the students’ marks to help them graduate. These behaviors not only severely undermine student quality but also affect the international reputation and status of Chinese higher education. There needs to be a system that deliberately holds back any student who is not mastering the knowledge and skills taught in the MBBS program.

## Conclusion and limitations

The findings of this study show that teaching-related factors play a critical role in influencing international medical students’ academic success. The problems identified here, when associated with other literature, suggest that the same problems are prevalent across other Chinese universities that host international medical students. It seems that although China MOE attaches great importance to development of MBBS education quality, there are still many issues in implementing its policies at the university level. These may be related to the importance and support each university attaches to the MBBS program, as well its own capabilities to teach the program effectively. The findings highlight that university policy-makers should devote more attention to the quality and development of MBBS education. Academics are the implementers of internationalization of education and they are the developers of educational resources ( Ding, 2016). Universities have the responsibility to support and meet the needs of academics in terms of professional learning and in provision of incentives, as well as being attentive to their recommendations for how to improve teaching quality. Efforts at individual level, department level, and university level should be jointly made to ensure high teaching quality, which is the key factor to promote student academic success.

This study was conducted at two sites, but caution with generalization of the findings is needed. They may be relevant to other Chinese universities offering courses to international students, but not to other settings. In future studies, this research will be extended to investigate the perceptions of academics on the salient *student-related* factors that may affect their academic success.

## Data availability

### Underlying data

The datasets generated and/or analyzed during the current study are not publicly available due to privacy agreements between the researchers and the interviewees. Making the interviews available – considering the relatively low number of international students at both Medical Universities – would likely compromise the anonymity of the respondents, thus breaking the ethical consent agreement that was established. Requests for data can be considered and if considered reasonable (e.g. having a professor position at a university) are available from the corresponding author Hugo Horta ( horta@hku.hk) or Qinxu Jiang (e-mail: jiangviolet86@hotmail.com). English language partial transcripts will be provided.

### Extended data

figshare: Interview protocol.docx. https://doi.org/10.6084/m9.figshare.20290566.v1 ( Jiang, 2022).

Data are available under the terms of the Creative Commons Attribution 4.0 International license (CC-BY 4.0).
