# Factors related to university teaching that influence academic success of international medical students in China

**DOI:** 10.12688/f1000research.123281.2

**Published:** 2024-02-12

**Authors:** Qinxu Jiang, Hugo Horta, Mantak Yuen

**Affiliations:** 1 Social Contexts and Policies of Education, Faculty of Education, The University of Hong Kong, Pokfulam Road, Hong Kong SAR, China; 2 Xuzhou Medical University, Yunlong District, Xuzhou, China; 3 Center for Advancement in Inclusive and Special Education, Faculty of Education, The University of Hong Kong, Pokfulam Road, Hong Kong SAR, China

**Keywords:** academic success, international medical students, China, medical education, university teaching

## Abstract

**Background:**

Academic success is extremely important for international medical students enrolled in Chinese universities, as it affects their performance in their licence exams and future work opportunities. However, insufficient research has been conducted on university academic staff’s awareness of teaching-related factors that affect their students’ academic success.

**Methods:**

A purposive sampling approach was taken in the study to recruit academics teaching in medical universities in China. The selection of informants was based on the subject they taught and their gender, experience of teaching international medical students, educational background and career stage. Semi-structured interviews were conducted with academics ( *N* = 36) between November 2020 and January 2021 at two medical universities in China. Each interview lasted between 30 and 70 minutes and was audiotaped, transcribed verbatim and thematically analysed.

**Results:**

The teaching factors that academics perceived to have an important influence on the success of students were (i) pedagogy and content alignment; (ii) language barriers; (iii) resource management and the learning environment; and (iv) educator attributes and guidance.

**Conclusions:**

University faculties and departments involved in teaching international medical students should ensure that their academic staff are supported in their ongoing professional development and provided with resources to enhance their teaching quality. The four factors (and their sub-factors) listed above should be prioritised in such staff training.

## Introduction

With the internationalisation of China’s universities over the past two decades ( Dang et al., 2023; Jin & Horta, 2018), medicine has emerged as the most popular programme, after the Chinese language, for international students to pursue ( Zhou, 2016). The Bachelor of Medicine and Bachelor of Surgery (MBBS) programme, taught in English, is designed and implemented by universities with the support and supervision of China’s Ministry of Education (MOE) ( Huang, 2014). An example is the document *Quality Control Standards of Basic Medical Education (in English Medium) for International Students in China*, which was published in 2020 by the China Education Association for International Exchange and the International Medical Education Committee, with the support of the International Department of the MOE. This document was aimed at further standardising MBBS education across Chinese universities and effectively improving training quality, thus promoting its sustainable development.

The MOE has authorised 45 universities to recruit international medical students ( Xie, 2021). The World Health Organization (WHO) recognises these universities in its Directory of World Medical Schools, which means that international graduates holding a Chinese MBBS degree are eligible to attend licence exams in their home countries. Examples include the Medical Council of India Screening Test, the United States Medical Licensing Examination, and the UK Professional and Linguistic Assessment Board. Students regard this as one of the greatest advantages of studying MBBS in China, in addition to other benefits such as less demanding admission requirements, lower costs, and a safe and stable society ( Zhou, 2016).

The MBBS programme typically takes six years to complete, with five years devoted to courses in natural science, the Chinese language, basic science medicine, and clinical subjects, and a one-year internship in a Chinese hospital or a hospital in the student’s home country. Most MBBS students are from low-income Asian and African countries, where the demand for health professionals is high ( Liu et al., 2017). The majority choose to return home to establish their careers after completing the MBBS programme, but some remain to pursue further studies ( Fan et al., 2020; Huang, 2019). Jiang and Sun (2007) found that 87.4% of MBBS students planned to develop medicine-related careers after graduation and hoped to make a significant contribution to the health workforce of their home countries, or planned to migrate to other high-income countries ( Han & Guo, 2009; Li & Sun, 2019). The quality of the Chinese MBBS education they receive is likely to influence their licence exam performance and their future employment positions. The reputation of these courses is therefore key to the sustainable development of China’s medical education for international students ( Zhou, 2016).

Medical licence exams are necessary to evaluate the teaching provided by medical schools and the learning outcomes of the graduates they produce ( Han & Guo, 2009). The success rate of MBBS graduates in these exams reflects the extent to which China’s MBBS education meets international standards ( Mao et al., 2012). However, the results have to date fallen been below those of several other countries. For example, more Indian students study in China than in any other country, and they are required to pass the Foreign Medical Graduate Examination (FMGE) to practise medicine in India ( Muthyanolla, 2022). The total number of Indian students who studied in China and sat the FMGE between 2015 and 2020 was 43,632, but the average pass rate was only 12.51% ( Muthyanolla, 2022). Similarly, for Nepalese students who studied in China, the pass rate in the Nepal Medical Council (NMC) Examination from 2016 to 2018 was only 34.3%, behind that of Nepalese graduates from Nepal (80.3%), the Philippines (74.8%), Bangladesh (60.9%), Pakistan (58.4%), India (49.2%), Russia (44.6%), and Ukraine (40.9%) ( Aryal, 2018). These results indicate that the majority of China-educated MBBS graduates do not have the expected level of professional knowledge or skills.

The selection of academics to teach international MBBS students is an important consideration. Priority is usually given to those with overseas educational or other experience, as this group of teachers are considered to be proficient in English and competent in intercultural communication ( Yu et al., 2022). To elevate teaching quality, Chinese universities also take measures such as sending academics for training in other countries ( Zhang et al., 2014). China’s MOE also established a Teacher Training Centre for studying medicine at Tianjing Medical University in 2010 ( Liu et al., 2021). This centre aims to improve the teaching standards and English teaching skills of medical teachers of international students in China and to improve the quality of training of international students and the level of education management services ( Liu et al., 2021). The centre takes a mixed-mode approach consisting of face-to-face training such as demonstration teaching, oral English training for foreign teachers, and online training such as lectures by domestic and foreign experts and teaching seminars ( Yu et al., 2023).

Although the successes of medical education for international students in China have been acknowledged in recent studies, several problems remain. These include the lack of a unified training programme for international medical students ( Liu et al., 2021b), language barriers between lecturers and students ( Ding, 2016; Huang, 2019), ineffective teaching pedagogy ( Yang et al., 2019a), insufficient qualified and experienced academic staff and poor teaching resources ( Ding, 2016; Huang, 2019; Wang et al., 2018), the lack of a robust quality assurance system ( Liang & Liu, 2019), classroom discipline issues ( Zeng & Sheng, 2019), and poor student quality ( Ma et al., 2018; Huang, 2019). However, many of these studies were based on the working experiences and subjective reflections of the authors, and thus they lacked empirical evidence. Some Chinese studies have taken a more objective quantitative approach by surveying international students and gathering their opinions, but the views of academic staff have rarely been solicited. The study reported here was designed to address this important issue by interviewing academics.

To the best of our knowledge, this is the first Chinese-focused study to investigate the beliefs of academic staff regarding the pedagogical factors that can contribute to the success of international medical students. The findings provide a better understanding of the challenges faced by international medical students in China and offer guidance for improving the quality of university teaching, to ensure that graduates have the necessary expertise and to maintain the reputations of the universities.

## Methods

### Ethics and consent

This study was approved by the Ethics Committee of Xuzhou Medical University (XZMU20200028) and the Institutional Review Board of Nanjing Medical University (NJMUIRB774) in September 2020. All of the informants gave written and oral informed consent to participate in the study.

### Research sites

The study was conducted in two medical universities (A and B) located in Jiangsu Province in eastern China, which has a well-developed economy and offers high-quality education. We selected these universities due to the availability of suitable academic staff and because they were representative of the MBBS programmes taught in other universities in the province. Both had accepted international MBBS students for over 15 years, so it was reasonable to regard them as relevant sites at which to gather the perspectives of academics engaged in teaching.

Both universities applied similar MBBS curricula, which followed the requirements of the MOE. International and Chinese MBBS students took separate courses and did not attend the same classes. English was the principal teaching medium of the courses for international students. Most of these students were from developing Asian and African countries, and some were far from proficient in English.

### Informants

We took a purposive sampling approach in this study. We based our selection of interviewees on the subjects they taught and their gender, years of teaching international medical students, educational background, and career stage. Our aim was to obtain a heterogeneous sample of medical teaching staff from the two universities. Academics were approached individually via phone calls and WeChat invitation with the help of two ‘gatekeepers’. Our target sample size before recruitment was approximately 30, but we increased this to 40 to achieve data saturation ( Morse, 1991). Of these, two declined the interview invitation and another two initially agreed but later withdrew by failing to respond to the researcher’s follow-up text messages.

Thus, a total of 36 academic teaching staff (17 men, 19 women) were recruited, of whom 2 taught Chinese, 3 taught nature science, 11 taught clinical subjects, and 20 taught basic medical science. Their experience of teaching international students ranged from 2 to over 13 years, and all were responsible for teaching the same subject to both Chinese and international students ( Table 1).

**Table 1.** Participants’ profiles.

Participant no.	Gender	Subject taught	Years of MBBS teaching	Academic rank	Overseas education/working experience
P1	F	Chinese	>11	Lecturer	None
P2	M	Anatomy	9	Professor	USA
P3	F	Community Medicine	>10	Lecturer	None
P4	F	Physics	>11	Associate professor	None
P5	M	Chemistry	>10	Professor	USA
P6	F	Biochemistry	>12	Associate professor	USA
P7	M	Physiology	>13	Lecturer	USA
P8	F	Immunology	>3	Professor	Japan
P9	F	Microbiology	>11	Associate professor	USA
P10	F	Histology & Embryology	>13	Associate professor	None
P11	M	Pathology	6	Associate professor	USA
P12	F	Pathoanatomy	>4	Lecturer	None
P13	F	Stomatology	>5	Lecturer	None
P14	F	Mathematics	>5	Associate professor	None
P15	F	Anesthesiology	>8	Professor	USA
P16	F	Physiology	>3	Lecturer	None
P17	F	Paediatrics	>12	Professor	Italy & USA
P18	M	Surgery	>12	Associate professor	Japan
P19	M	Human parasitology	>8	Associate professor	France
P20	M	Diagnostic radiology	>2	Associate professor	Hong Kong
P21	F	Cell biology	>10	Associate professor	USA
P22	M	Pharmacology	>2	Associate professor	Japan
P23	F	Diagnostic radiology	>2	Associate professor	Hong Kong
P24	M	International medicine	>13	Associate professor	UK
P25	F	Diagnostics	>5	Lecturer	None
P26	M	International medicine	>13	Professor	Germany
P27	F	Obstetrics & Gynaecology	>13	Associate professor	None
P28	M	Anatomy	>14	Professor	USA
P29	F	Human parasitology	>10	Associate professor	USA
P30	F	Hygienics	>6	Associate professor	USA
P31	M	Pharmacology	>2	Lecturer	USA
P32	M	Chinese	>9	Associate professor	USA
P33	M	Paediatrics	>18	Professor	USA & Canada
P34	M	Surgery	>11	Professor	None
P35	M	Surgery	>6	Associate professor	UK & USA
P36	M	Physiology	>8	Professor	Singapore & USA

### Data collection

A semi-structured interview was conducted with each participant between November 2021 and January 2022. Only the first author and one participant were present in each interview. Academics from University A participated in face-to-to-face interviews in their offices or a small meeting room that was perceived by the participants to be comfortable and quiet, while the interviews with academics from University B were conducted online (due to the COVID-19 pandemic). Both the face-to-face interviews and the online interviews were audio-recorded and notes were taken throughout. The interview questions, which were pilot-tested, addressed the participants’ views on factors affecting student success, and their recommendations for improving teaching quality at the classroom and organisational levels.

The academics were initially briefed about the probable length of the interviews and the purpose and significance of the study. To conform with ethical requirements, all interviewees were assured of their anonymity and agreed to the audio recording and dissemination of the findings. Each interview was conducted in Chinese and lasted for around 30 to 70 minutes.

### Data analysis

A thematic approach was taken to the data analysis ( Braun & Clarke, 2006), with the audio recordings later transcribed verbatim. These Chinese transcripts were coded manually by the primary researcher, who immersed herself in the data and noted down her initial ideas on emergent themes. Initial codes were generated based on identified key words and phrases. These codes were then summarised into potential themes. The primary researcher translated the codes and themes into English. The research team then discussed, adjusted, and reached a consensus on a final set of categories. The integrity of the process was ensured through member checking, peer auditing and self-reflection. The primary researcher kept a self-reflection journal throughout the process to minimise the effects of her own background and work experience on the interpretation of the data.

## Results

### Theme 1: Pedagogy and content alignment

***1.1 Challenges in teaching pedagogy***

Most of the academics perceived their teaching methods and practices to have a major influence on international students’ learning. However, they perceived a mismatch between their teaching approaches and the learning characteristics of these students. They mainly relied on traditional teacher-centred lecturing and tended to read verbatim from slides, without giving adequate further explanations or offering practical examples that could enhance the students’ comprehension of the subject. Some noted that as a learning approach, lectures may not be suitable to meet international students’ needs. A teacher of clinical medicine commented that in a lecture on clinical manifestations and diagnoses of diseases, the students were primarily concerned about the treatment and recovery aspects. The teacher suggested:

*The students enjoyed learning about clinical treatments maybe because they thought that it would be useful for their clinical work in the future. However, treatment is only briefly introduced in this class.* (P25)

International students asked more questions than Chinese students and were willing to interrupt the class to express their opinions. This led the academics to consider how they could better communicate with them and how to improve the quality of their teaching. Most felt that much of the current teaching involved spoon-feeding information and lacked interaction. Lecturers were failing to grasp students’ attention and hold their interest in class, which often affected their exam performance. Interviewees felt that teaching innovations such as case-based learning (CBL) and problem-based learning (PBL) should be used to teach international students, because this type of teaching mode is more heuristic and thus encourages students to learn more actively. They suggested that CBL and PBL would increase the involvement of international students in learning activities. They could also make more frequent use of individual and group presentations and discussions, encourage students to ask questions, guide students to think, and try flipped class teaching (setting preparation assignments on a lecture topic before presenting the lecture). However, the academics also felt that implementing these changes would be a challenge for them, because it would place more demands on their language skills, subject knowledge, ability to listen and respond to students, and control the class. One of the few academics who already involved international students in interactive class learning activities reported positive changes in the performance of these students:

*International students are not interested in the traditional lecturing we use to teach Chinese students. Very few students listen carefully in class. I usually ask international students to summarise the content of the previous lesson in front of the whole class. They participate very actively and prepare well. I think international students like to show themselves in public. If I take advantage of this characteristic of theirs, I can stimulate their passion for learning.* (P31)

International students’ basic knowledge and learning were poorer than those of Chinese students. The lecturers acknowledged the diverse academic abilities of international students and their different cultural backgrounds and agreed that they should adjust their teaching approaches accordingly. They also believed that they should give culturally relevant examples in class that the students were familiar with, make timely summaries of key knowledge points, give them practice assignments to complete after class, cover exam points, and provide timely feedback on their assignments. An academic teaching radiology gave the following example:

*For international students, you have to start from the basics. For example, in my [radiology] class, there is a CT value, high or low. High is bright, low is dark. You have to explain this foundation setting first; otherwise, they would become confused. Doing this sort of thing could help them but would require academics to take more time to prepare for class.* (P19)

The interviewees reported that real clinical cases should be integrated into basic medicine lectures to help international students understand them, and they thought that the students would learn better with case examples. Academics without extensive clinical experience but who taught biochemistry, microbiology, immunology, and parasitology found this to be a challenge. They said that they had to provide more than theory and facts and include relevant clinical knowledge to properly teach their students. An academic teaching biochemistry who had a background in biology stated as follows:

*Students have asked me some clinical questions, which shows that they have this demand. This demand actually reminds me that I should include clinical knowledge in my teaching. This could make my class more attractive and make them realise that my course is very helpful for their clinical study in the future and can help them solve clinical problems. In addition, it can make students realise the importance of my course, at least for now, and increase their learning enthusiasm. I’ve already started learning relevant clinical knowledge.* (P6)

Some of the academics also acknowledged that their lecture slides probably needed to be more interesting. They stated that it may be necessary to use teaching aids in addition to writing on the blackboard, such as video, audio, and animation, to make the classes more dynamic and easy to understand for students. An academic teaching pharmacology mentioned how he used online technology to make the learning in his class more effective.

*I like to visit YouTube to find some videos about the mechanisms of drugs to show in the class. Maybe my explanations are not very good, but the videos explain very well, which can help deepen students’ understanding. Moreover, I feel that playing videos when students are sleepy can increase their attention.* (P22)

***1.2 Alignment of teaching content with licence exams***

Over a quarter of the interviewees noted that a primary reason why many international students lack interest in learning is that the current course content and resources do not correspond closely enough to the requirements of the licence exams. Academics usually teach the knowledge that they think is important, and this may not precisely match a curriculum based on the licence exam. A combination of academics’ specialist knowledge and a prescribed curriculum is required. This is a critical issue, as students will be more motivated to learn if they perceive that what they are asked to learn will be useful for passing examinations and in their working lives. However, some of the academics interviewed argued that it would be difficult to address all of the licence exam needs of students from diverse countries, as the content is likely to vary. Developing a more contextualised curriculum therefore seems essential, as explained by an academic teaching anatomy:

*One of the key points of our teaching is to help students pass licence examinations. If a student does not pass the exam, he/she will not even qualify for matching hospitals. This is true for both Chinese students and foreign students. Teaching and licence examinations cannot be separated. If we want to help students pass the licence exam, teaching activities should play a guiding role … The passing rate for licence exams is the most important standard measure of a medical university. Practice is the sole criterion for testing truth.* (P2)

### Theme 2: Language barriers

In addition to teaching pedagogy, language barriers were identified as a significant challenge to successfully teaching international students. Most of the interviewees indicated that language differences and difficulties negatively influenced their teaching and students’ learning quality. They also offered various potential solutions. These Chinese academics spoke English as a foreign language, while international students spoke English as their first or second language (although many were far from perfect in English). This is not a criticism of either group, but merely reflects the actual situation. Communication was therefore limited because the academics were not necessarily fluent in English, and neither were many of the students. The academics often found it difficult to understand their English accents and expressions, as they may not speak standard American or British English, particularly those from India, Nepal, and Pakistan.

The academics admitted that while they generally had a good command of medical English in their own field after years of teaching, they still struggled with day-to-day and cross-disciplinary English. This limited vocabulary outside of their field affected their teaching and was one of the main reasons why they often resorted to reading verbatim from slides rather than explaining concepts in their own words, and why they avoided engaging with students. Consequently, their students become bored during class and were sometimes annoyed by the poor communication. One academic who taught surgery noted the difference between Chinese and international students in terms of language in theory lectures.

*If it is a Chinese class, the academic has many methods of teaching. He has eloquence and rich experiences; he can solicit and quote from others when giving lectures. However, in case of teaching foreign students, due to the language barrier, academics cannot express themselves 100% smoothly. They can only communicate with students in the simplest way, so they cannot always respond appropriately to students. If they cannot attract the students, the students will lack interest in the class. Naturally, they do not devote themselves to learning.* (P35)

Academics teaching clinical medicine who were also responsible for clerkships and internships at hospitals indicated that it was hard to translate some Chinese words concerning a patient’s symptoms into English. Sometimes the translation was not completely accurate, and international students could not comprehend explanations delivered in conversational Chinese. Therefore, the effectiveness of clerkship and internship learning in clinical medicine was adversely affected because of language barriers, as one academic teaching internal medicine explained:

*Take the symptoms of angina pectoris for example. When I talk about angina pectoris to Chinese interns, I use the Xuzhou dialect, which is very easy to understand. However, it is difficult for me to convey the clinical symptoms reflected by most patients to foreign students through a more appropriate word, because the pain reflected by the patient is not the oppressive feeling mentioned in the textbook. We cannot simply transfer the knowledge from books to students; we have to combine it with our clinical practice. However, sometimes, it is very difficult to translate properly.* (P25)

### Theme 3: Resource management and learning environment

***3.1 Management of teaching resources***

Over half of the interviewees stated that issues with teaching resources also affected teaching and learning. Four sub-issues emerged within this theme, as follows.

***3.1.1 Demand of textbooks and teaching materials***

Twelve academics complained that they lacked easy access to necessary textbooks, English question banks, and other relevant teaching materials, such as reference books and videos. Universities did not mandate or provide any particular textbook for academics to use, and the academics had to seek out textbooks or create their own materials by drawing on various English and Chinese sources. If the textbooks were different from the students’ own, they could find it difficult to understand the lectures. The students were at times not assigned any textbooks or relevant learning materials. In addition, academics responsible for different modules or units in a course often used different textbooks, which led to inconsistencies. Thus, they felt that experts should develop good textbooks to help students understand and review the knowledge learned in the lectures, and they suggested that universities should purchase specialised English books, medical videos, and question banks for staff and students to use. One academic teaching gynaecology commented on her difficulty with using textbooks as follows:

*Our Clinical College once gave us some textbooks, but I think the versions of those books were relatively old. I have not seen updated books. In those books, old methods of screening gynaecological cervicitis, which were used one or two decades ago, were introduced.* (P27)

***3.1.2 Need for collaborative lesson planning***

Seven academics felt that it was important to engage in collective lesson planning to ensure effective communication. This approach is currently lacking, but can be important for a teaching team to share experiences and discuss which knowledge points are the most important for students to master. These planning sessions can be used to discuss the application of flipped class teaching and how to properly create synergies among different topics taught by different academics. Experienced academics could also advise new members of staff about how to teach international students through collective lesson planning. Those inexperienced in teaching international students expressed their need to receive teaching tips about appropriate pedagogies, and to obtain feedback from more experienced lecturers. However, in most departments, and particularly in clinical medicine subjects, collective lesson planning and guidance was lacking, as the teachers were busy with their own clinical work and research:

*Theoretically, there should be collective lesson planning, but it cannot be done in practice … Teachers have their own interpretations of knowledge and lecturing methods, and collective lesson preparation has little impact on them … Basically, teaching has been weakened and become a mere formality. There is no teaching demonstration for new teachers anymore. The supervision mechanism has gradually disappeared. Now the overall orientation no longer takes teaching as the main evaluation index, but mainly takes SCI publications.* (P24)

***3.1.3 Weak teaching supervision system***

Seven academics reported that universities lacked a sound system for supervising teaching quality. This may lead academics to conclude that it makes no difference whether their teaching is of a good standard. Without a system that rewards good performance and penalises poor performance, academics have no incentive to teach well. Some of the interviewees stated that strengthening the supervision of teaching through regular observation by experts and spot-checking lecture quality would benefit academics.

*The School of International Education needs to establish a system or assessment standard and implement rewards and punishments in each teaching and research department. If there is no difference between good and bad teaching, teachers will not be motivated.* (P33)

***3.1.4 Inadequate clinical teaching resources***

Five senior clinical academics mentioned that there was a shortage of well-qualified personnel to teach theory, clerkships, and mentoring internships in hospitals. Due to the high turnover of staff, some departments lacked a stable team responsible for teaching international students. They were often taught by recently hired academics or new doctors who had minimal or no prior teaching experience. Their approach was limited to reading their notes or slides out loud, without any explanation or discussion. Those with teaching experience felt that universities and hospitals should attach more importance to consistency in teaching international students, and should introduce incentives to encourage young, qualified doctors to participate in teaching. A perceived need to train new academics for future teaching work was observed:

*During the clerkship, we had two teachers to teach 25 international students. The venue space and teachers were not enough … For foreign interns, an internship was more like clerkship learning, just for a visit, because some teachers could not speak English and they were very busy. Foreign interns could not operate, due to doctor–patient relationship concerns. They could not receive much guidance … You know, this is a problem.* (P24)

Three academics also mentioned that their course syllabuses were obsolete. A senior academic who taught paediatrics said it was imperative to discuss with international students which specific diseases were common in their own countries, and thus what they needed to learn about. The course syllabus could then be updated accordingly. The students could then more easily relate to the content being taught in class. These academics suggested their departments should evaluate their existing syllabuses and improve them by making them more relevant where necessary.

***3.2 Classroom discipline management***

Chinese academics felt frustrated and perplexed by some of the behaviour of international students in class. These students were often late to class, left in the middle of the class for no reason, or listened to music, used smartphones, and chatted with others and made noise during class. The mood of the teaching staff and their attitudes towards international students was adversely affected by such behaviour, as such discipline problems would rarely occur in classes of Chinese students. Seven academics stated that although such behaviour might be due to cultural differences, the students should follow Chinese rules. The academics dedicated time to disciplining the students to make them aware of the seriousness of their studies. However, some emphasised that it was the responsibility of the student administrators to properly manage student discipline. One academic teaching hygiene explained how she managed class discipline and encouraged students to learn.

*In sharp contrast to Chinese students, foreign students pay too little attention to class discipline... For example, for morning class at 8 a.m., some students do not come until 9 a.m. I wondered why. Later, I went to Pakistan for a visit. When I went to a university, I understood that this was their habit. However, in China, if you want to obtain a Chinese degree, you have to follow Chinese rules … I count the check-in time and lateness in the class participation score, and I call the roll five minutes in advance. Sometimes I close the classroom door for fear that they might sneak out. Sometimes I take a roll call in the second quarter of class to prevent anyone from sneaking out after signing in … It is like fighting a battle of wits and of courage with them.* (P30)

***3.3 Assessment methodologies***

Six academics stated that the current assessment system was problematic, and they specifically mentioned the exam evaluation system and the final elimination system.

***3.3.1 Exam evaluation system***

Only a summative assessment based on final exam performance was conducted at the two focal universities, with no formative assessment. Some of the participants suggested that the university should add formative assessments such as a mid-term exams or in-class quizzes, because this could help academics follow students’ progress, identify any difficulties, push students to study harder, and reduce the pressure they felt regarding the final exam.

A teacher of pathophysiology suggested that any evaluation of international students’ academic performance should take into account their specific characteristics rather than strictly following the approach for Chinese students. This academic also felt that international students were more interested in practical learning in laboratory classes and generally outperformed Chinese students in these classes, so the university could consider increasing the weight of the practical score in their final course scores.

Some also mentioned they wanted to change the assessment methods, but in most cases, this was beyond their control, as the changes had to be approved at the organisational level after going through complicated application procedures. One participant who taught parasitology noted how their department had improved the assessment method based on students’ feedback.

*Students came to complain that the exam was unfair because some students did not attend class, just memorised key points the week before the exam and got higher marks. After receiving their feedback, we improved this. We took their formative performance as a part of their assessment, and the final exam accounted for only 50%. Answering questions and signing into class are all part of the formative performance. I think we should add formative assessment and tell students to listen to the whole class. Our purpose is not to let them fail in the exam, but to guide them to learn something. Students understand this. Finally, almost all students passed my course.* (P29)

***3.3.2 Final elimination system***

Seven academics pointed out that the universities’ assessment and evaluation systems were less strict for international students. This was perceived as a loose approach to entering and exiting the course, and did not put pressure on students to study. These academics suggested the university should implement a final elimination system to delay graduation or fail those who do not have the ability to graduate or attain an MBBS degree.

*The university must strictly enforce the examination system and cannot deliberately make exceptions in international students’ favour. Schools should dare to weed out unqualified students, so that they will be afraid. Once they are afraid, most of them will study hard.* (P10)

### Theme 4: Teacher attributes and guidance

***4.1 Addressing teacher attributes***

Nearly half of the interviewees acknowledged that the attributes of academics, such as their dedication, passion for teaching, positive attitude towards teaching, sense of responsibility, and subject knowledge, play a pivotal role in ensuring teaching quality and thus enabling students to learn. They suggested that much more time and effort is needed to prepare lessons for international students, due to the modifications required. Some regarded the workload of preparing a lesson in English to be three to ten times greater than preparing a lesson for Chinese students. For instance, they had to write a lesson script beforehand to read aloud in class, because it was too challenging to improvise in English during the session. This type of language problem would not occur in Chinese classes.

The interviewees admitted that many academics were not motivated to teach international students because their universities prioritised research over teaching. Academics had no incentives to devote time and effort to areas that would not advance their careers. Therefore, although they were qualified to teach international students, they felt more motivated to conduct research. Apathy regarding teaching also affected its quality. A typical observation was as follows:

*All materials for lesson preparation must be in English. It is already very difficult to prepare lessons in Chinese. I have to prepare lessons in English and practice my manuscript … In this current university environment, which generally attaches importance to scientific research rather than teaching, everyone is willing to focus on their own research rather than pay attention to teaching. Research awards are more tempting... When it comes to professional rank evaluation, papers are more useful than a teaching competition award.* (P3)

Despite the common perceptions of a heavy workload and challenges and dissatisfaction due to the lack of incentives, some academics maintained a personal commitment to their teaching out of a sense of responsibility and self-improvement.

*To maintain the enthusiasm of academics for teaching, it is not about monetary stimulation, but a sense of value and honour. Academics are not unsentimental people … Many academics are dissatisfied, but they continue to teach even if they are not satisfied. This is dedication, awareness, and sentiment … I don’t want to talk about interests, but the school should give me some other aspects that emotionally make me feel honoured to teach international students.* (P4)

When asked about what incentives universities could provide to motivate academics, the interviewees said that university management should attach more importance to MBBS education and to the academics who provide it, instead of regarding the field as peripheral. They emphasised the need for continuous professional learning to improve pedagogies, and assistance with oral English through formal or informal learning opportunities at home or abroad. Several academics suggested that their teaching performance should be regarded by the university as part of its evaluation for promotion, rather than solely basing it on the number of publications. This would motivate academics to teach and make them more enthusiastic about teaching. A few academics also said they had sought funding so they could implement teaching innovations in the MBBS programme, but had been unsuccessful. Two academics felt that the university should count their class hours as double or three times more than for Chinese classes, because they spent much more time on lesson preparation and class hours were considered in terms of academic promotion.

***4.2 Supervision and guidance by teachers***

When asked what academics could do to help students improve their performance, nearly half of the interviewees indicated that there was a need for them monitor their students’ progress more closely, and to push underperforming students harder, to make them aware they were responsible for their own learning. Some students were lazy and lacked self-discipline. The academics felt that if they could devote more time to guiding low-achieving students and talking with them after class to encourage them, students might feel that their teachers are concerned about them and become more motivated to study. An academic teaching cell biology highlighted the importance of encouragement and caring about students:

*Students are still children. If academics communicate with them more, they will feel pressure and study under pressure … In the first year of my teaching, I dedicated all my energy to push them. I tried my best to memorise all of the students’ names and faces. They felt my care. Several students have kept in contact with me since graduation … I was busy with my own life and family, but if I had spent more time at that time, I could have saved a few more students from failing.* (P21)

Six academics felt that it is important to impart not only knowledge and skills to students but also moral and ethical education, values, and a sense of responsibility. They stated that it is critical to teach students to cherish learning opportunities in China, to ensure they have the correct learning attitudes, and to acknowledge their role in medicine. This would stimulate students’ intrinsic motivation and instil in them a sense of respect and responsibility, as stated by an academic teaching anatomy:

*In anatomy class, the specimens are donated by others. No matter which country in the world, the donors are those worthiest of respect. As a medical student, you should respect them and the patients, because they, ordinary people, have given us the opportunity to learn and explore medicine. You should treat patients well in the future. Why did they donate their bodies? Because they hoped you could become a good doctor.* (P28)

***4.3 Establishment of teacher–student rapport***

One third of the interviewees mentioned that getting to know students and their cultures and establishing good teacher ***–*** student rapport are conducive to better communication and teaching. Good communication between academics and students can help build emotional connections and understanding in the classroom. For example, without knowledge of students’ lives and cultures, an academic might accidentally be culturally insensitive and cause discomfort. An academic teaching orthopaedics with more than ten years’ experience of teaching international students shared how he successfully developed a good relationship with students by incorporating aspects of their culture into his class.

*Once, as soon as I finished watching the Indian film* Dangal *, I quoted ten classic sentences in my slides to motivate my students in class. They became very interested when they saw these quotes. “Wow, this is our Indian movie.” Sometimes, I talk about my feelings about Nepalese festivals in class. They feel excited and think the teacher acknowledges their culture... If students like you, they will learn your course well.* (P18)

An academic teaching anaesthesiology mentioned that those who taught clinical subjects generally knew less about their students, and thus they failed to establish a good rapport. This made their lectures seem rigid and lacking attention to the students as individuals. Those who taught clinical medicine suggested that universities should arrange activities to increase communication between staff and students, which is particularly important for younger academics.

## Discussion

From the perspectives of academics who teach international students, this study revealed the critical factors that can affect the academic success of international students in China. Four main themes emerged: the challenges associated with pedagogy and content alignment, language barriers, resource management and the learning environment, and educator attributes and guidance.

Pedagogy and language barriers were the factors most frequently reported to affect Chinese academics’ teaching effectiveness and international medical students’ academic success. This supports the findings of the systematic review by Dang et al. (2023), who identified these factors as the most common challenges facing Chinese academics in English-medium-instruction (EMI) programmes. In addition, Helm and Guarda (2015) reported that in an Italian university, most lecturers of programmes where English is the medium of instruction needed to improve their teaching competences, which echoes our finding that pedagogy as the factor perceived most relevant to student academic success. The literature also indicates that the pedagogical skills of academics are significantly influenced by language barriers ( Ding, 2016; Huang, 2019; Zhan, 2017). Yang et al. (2019b) found that EMI teachers in a Chinese university were more didactic and less interactive in class than non-EMI teachers, which then hindered teaching and learning. However, improved English does not necessarily equate to improved pedagogy ( Dafouz, 2018; Dang et al., 2023; Helm & Guarda, 2015). The findings of this study suggest that while it is important for universities to address the oral English abilities of academics in terms of both specific disciplines and in daily life, improving their pedagogy is more important, as this is key to the academic success of international medical students. Universities should offer ongoing professional learning through teaching approaches such as PBL and CBL for pre-service and in-service academics, and they should fully consider the needs, abilities, and interests of international medical students ( Liu et al., 2021b; Saleh et al., 2012; Yang and Lei, 2021). Universities should also strongly encourage and support teaching innovations with sufficient funding.

The problem of the detachment of teaching content from the licence exam requirements has been recognised in some Chinese studies ( Han & Guo, 2009; Huang, 2019). Some universities have been able to integrate licence exam content into their teaching. For example, the MBBS curriculum at Zhejiang University has been internationalised with reference to UK medical education standards and clinical executive standards such as Tomorrow’s Doctors ( Fan et al., 2020), the United States Medical Licensing Examination, and the Professional and Linguistic Assessment Board. Li and Cui (2010) from Dalian Medical University suggested introducing licence exam tutoring by inviting foreign academics to teach Chinese academics using exam questions and content, but this was viewed as separate from regular teaching. This implies that university staff should not only adequately prepare students for assessments but also shape the curriculum to include relevant topics (e.g. topics relevant to the FMGE).

This study revealed that limited access to teaching resources can damage the teaching and learning experience. The lack of standard English textbooks and relevant teaching materials for international students has been noted in many Chinese universities ( Huang; 2019; Jiang et al., 2014; Yang et al. 2019a; Zeng & Sheng, 2019) and in other countries, such as Ukraine, where EMI programmes are taught to domestic university students ( Goodman, 2014). Subject content is acknowledged to be mainly delivered through textbooks, so their selection and use will directly affect teaching and students’ knowledge attainment ( Li & Cui, 2010; Li et al. 2013). Compared with the rich integrated curriculum teaching resources in developed countries such as in Europe and the US, China lacks English medical teaching materials that have complete independent intellectual property rights and that align with the characteristics of its medical education in terms of relevance and adaptability ( Wang et al., 2018). The dire shortage of English teaching resources has limited the sustainable development of medical education for international students in China ( Wang et al., 2016). Although some Chinese universities have begun to write their own textbooks and have made progress, problems remain. For example, Chinese teachers’ levels of English ability are uneven, and their writing proficiency lacks unity ( Wang et al., 2018).

In alignment with the literature ( Ding, 2016; Huang 2019; Yang et al., 2019a; Zeng & Sheng, 2019), the findings of this study show that a lack of collaborative lesson planning for MBBS classes, a shortage of clinical teaching resources, and weak supervision mechanisms are common in Chinese universities. In terms of the perceived obsolete syllabuses, Zhou (2016) noted that Tianjin Medical University has added relevant teaching content according to changes of the disease spectrum in the country of origin. For instance, *Schistosoma aegypti* and *Schistosoma mansoni*, which are prevalent in Africa, were added to the Human Parasites course. The example of Tianjin Medical University implies that organisational support plays a critical role in guaranteeing the quality of MBBS education.

Classroom disciplinary issues are common with international students and can negatively influence the lecturing style of academics. This problem has also been identified in other studies ( Jin et al., 2009; Zeng & Sheng, 2019). Some academics take disciplinary action, while others ignore the behaviour of problem students but feel uncomfortable with the lack of control. In a survey of learning experience in a medical university in Southwest China, half of the MBBS students felt that the academics were not strict enough in class ( Yang & Lei, 2021), probably because of the language barrier. However, the findings of this study suggest that academics should ensure good disciplinary management to encourage good behaviour amongst students. Action needs to be taken with misbehaving students so they focus on attendance and listening during lectures.

The findings regarding the influence of assessment methodologies on international medical students’ academic achievement have rarely been discussed in the literature. In a study conducted in Ningxia Medical University, Zhang et al. (2020) focused on how the implementation of formative assessment in its MBBS microbiology course helped improve class attendance and learning attitude, develop creativity and self-directed learning habits, increase teacher–student communication, and made evaluation fairer and more objective. This demonstrates the advantages of revising assessment styles. Zhao et al. (2020) claimed that the current assessment of students in medical colleges and universities in China is generally summative (at the end of the course), and that there is a lack of understanding of and attention to formative (ongoing) assessment and evaluation during the course. Summative assessment was viewed by the interviewed academics as a relatively single and weak method that could reduce students’ enthusiasm for learning. Assessment only at the end of a course makes it difficult to monitor progress in students’ professional knowledge development ( Zhan, 2017). Therefore, universities should appropriately adjust their assessment methodologies to encourage international medical students to learn and improve the learning processes and environment.

As in other studies ( Wang, 2013; Zhang & Hu, 2019), we noted that there was often a tendency in Chinese universities to graduate international students by reducing the level of exam difficulty or lowering the pass mark, or by modifying the students’ marks to help them graduate. These approaches not only severely undermine student quality but also affect the international reputation and status of Chinese higher education. A system that deliberately holds back any student who is not mastering the knowledge and skills taught in the MBBS programme is required.

Blömeke et al. (2016) found that attributes of academics such as their experience, education background, motivations, professional knowledge, and self-efficacy predicted student achievement. Similarly, our findings suggest that academics’ characteristics, such as passion for teaching, professional responsibility, dedication, attitude, and content knowledge, can have an effect on teaching quality. As indicated by the interviewees, academics are often demotivated to teach international students because of a lack of emotional, material, and career incentives. A lack of incentives seems to be a prevalent phenomenon, as reported in other universities in different provinces in China, such as Liaoning Medical College ( Ding, 2016), Three Gorges University ( Zeng & Sheng, 2019), and Zhengzhou University ( Zhang, 2016). The problem has also been identified in other countries such as Sweden ( Stenfors-Hayes, 2010). This suggests that university policy makers need to be attentive to the opinions and needs of academics when developing an appropriate reward system that can promote teaching quality and thus student success.

The findings concerning teacher guidance for students and its effect on their success is new to the literature. Chinese academics appeared to subconsciously assume a parental role when disciplining international students, which implies their commitment to education. The importance of providing moral and ethical education for international students during and outside lectures has also been recognised in Chinese studies ( Liu et al., 2021a; Wang & Huang, 2019). This may be particularly relevant in a Chinese context because of the influence of Confucian ideology on Chinese education, which suggests that academics should not only impart knowledge and skills but also have an obligation to cultivate the mind ( Ye, 2001).

As previous studies set in China ( Yang & Lei, 2021; Zhao, 2021) and Iraq ( Saleh et al., 2012) have noted, this study revealed that teacher–student rapport is an overlooked dimension in MBBS teaching. Differences in language, cultural background, and religious beliefs can lead to a lack of such rapport, and make the dynamics within a classroom dull and rigid ( Yang & Lei, 2021). Building a good relationship between academics and their students may help to foster a more favourable classroom environment for learning. This will require academics to learn more about the life and learning characteristics of international students and to communicate with them more after class to establish a harmonious and supportive teacher–student relationship.

## Conclusion and limitations

The findings of this study show that teaching-related factors have a major influence on international medical students’ academic success. The problems identified, and the findings of other studies, suggest that the same problems are prevalent across Chinese universities that host international medical students. It seems that although China’s MOE attaches great importance to the development of MBBS education quality, many challenges remain to implementing its policies at the university level. These issues may be related to the importance and support that each university attaches to the MBBS programme, and its own ability to teach the programme effectively. The findings highlight that university policy makers should devote more attention to the quality and development of MBBS education. Academics are responsible for implementing internationalisation in education and the developers of educational resources ( Ding, 2016). Universities have the responsibility to support and meet the needs of academics in terms of professional learning and in the provision of incentives, and they should consider their recommendations for improving teaching quality. Effort should jointly be made at the individual, department, and university levels to ensure high teaching quality, which is the key factor in promoting student academic success.

Our study has several limitations. It was conducted at two sites, so caution should be taken when generalising the findings. They may be relevant to other Chinese universities offering courses to international students, but not in other contexts. Both research sites were located within a single province, and although representative of the general MBBS education in other universities in the same province, a broader geographical scope or the inclusion of more diverse medical universities from other regions or provinces might have led to new or different insights. Additionally, as the data were sourced from subjective opinions expressed by the academics, potential biases may exist. In future studies, this research could be extended to investigate the perceptions of academics on other student-related factors that may affect their academic success.
