# Supplementary material for: Factors related to university teaching that influence academic success of international medical students in China
Source: F1000Res. 2024 Feb 12;11:894. Originally published 2022 Aug 4. [Version 2] doi: 10.12688/f1000research.123281.2 (PMC11109579; doi:10.12688/f1000research.123281.2)
Supplement: Peer review history

# Reviewer report: Reviewer response for version 2

**DOI:** 10.5256/f1000research.162163.r257479

**Published:** 2024-05-07

**Authors:** Masego B. Kebaetse

**Affiliations:** 1 University of Botswana, Gaborone, Botswana

Overall, the manuscript is clear and well-written. The gap within the specified context is clear. Here are some suggestions to improve the manuscript.

**Introduction:**

 1] para 1, line 5 - kindly revise the sentence starting with "an example". For instance, something like this 

 "The Quality Control Standards of Basic Medical Education (in English Medium) for International Students in China document was released in 2020 by the International Medical Education Committee, the China Education Association for International Exchange, and the MOE's International Department with the goal of further standardizing MBBS education across Chinese universities and improving training quality."

 2] It seems that para #2 could move to line 5 of para#1 just before the revision above.

 The Ministry of Education has authorized 45 universities to recruit international medical students (Xie, 2021). The World Health Organization (WHO) recognizes these universities in its Directory of World Medical Schools, allowing international graduates with a Chinese MBBS degree to take license tests in their respective countries. These include the Medical Council of India Screening Test, the United States Medical Licensing Examination, and the UK Professional and Linguistic Assessment Board. According to Zhou (2016), studying MBBS in China offers numerous benefits, including shorter entrance standards, lower prices, and a safe and stable society. 

**Methods:**

 1] kindly include the study design before the ethics section

 2] consider "we used purposive" vs. "we took" a purposive sampling

 3] consider "semi-structured interviews were conducted between ..."

 - were all the interviews conducted in Chinese? Please indicate the languages used (I may have missed this).

 The data analysis needs clarity, not necessarily long.

 4] Consider: "Data analysis was conducted using the Braun and Clarke (citation) framework to identify patterns in the data. Audio recordings were transcribed verbatim." 

 - what type of verbatim transcription did you use" Please indicate and provide a citation.

 -what do you mean by coded manually? 

 - were the Chinese recordings transcribed and translated into English, or did you only translate what you needed for the manuscript?

 - when you say, "Initial codes were generated based on identified keywords and phrases" what do you really mean?

 If you say that you used Braun and Clarke, the description of the data analysis should be consistent with the important components of the framework.

**Results:**

 1] It would have helped to see the naming of the participants include institutions A or B so readers can have a sense of the distribution of participants in the data extract

 2] the authors tend to provide long summaries and then opt for long extracts at the end of the paragraph. Shorter data extracts can be included in the summary to ensure that the summaries are grounded in the data. The 22 data extracts come from only 22 of the 36 participants.

**Discussion:**

 OK, though it seems to be missing the "so what."

Is the work clearly and accurately presented and does it cite the current literature?

Yes

If applicable, is the statistical analysis and its interpretation appropriate?

Not applicable

Are all the source data underlying the results available to ensure full reproducibility?

No

Is the study design appropriate and is the work technically sound?

Yes

Are the conclusions drawn adequately supported by the results?

Yes

Are sufficient details of methods and analysis provided to allow replication by others?

Partly

Reviewer Expertise:

academic success and learning challenges, first-year experience, elearning, curriculum development and support, faculty development.

I confirm that I have read this submission and believe that I have an appropriate level of expertise to confirm that it is of an acceptable scientific standard, however I have significant reservations, as outlined above.

# Reviewer report: Reviewer response for version 2

**DOI:** 10.5256/f1000research.162163.r246044

**Published:** 2024-02-16

**Authors:** Myla Arcinas

**Affiliations:** 1 Department of Sociology and Behavioral Sciences, De La Salle University, Manila, Metro Manila, Philippines

The authors carefully considered the reviewers' suggestions in the revised version of the manuscript, which resulted in significant improvements to the study's depth and clarity. Particularly commendable is the enhanced elucidation of the process by which academics at Chinese medical universities are selected and assigned to teach international students. The clarification that international students are segregated from their Chinese counterparts in both classrooms and residential arrangements adds crucial context to the educational environment under investigation.

 Furthermore, the authors have augmented the description of the research sites and provided comprehensive insights into the characteristics of the participants, including pertinent details such as gender, academic rank, and educational background. This enriched characterization enhances the readers' understanding of the study population and adds nuance to the analysis.

 Noteworthy revisions to the findings section, including the reorganization and refinement of thematic designations, demonstrate a commitment to clarity and precision. The expanded discussion on the alignment of teaching content with licensure exams reflects a deeper exploration of a key aspect of the study, thereby enhancing its relevance and applicability.

 Moreover, the authors have adeptly expanded the discussion to encompass pertinent issues such as classroom discipline and the adaptation of teaching methodologies to accommodate diverse international student populations. This broadened scope not only enriches the discourse but also underscores the practical implications of the study findings.

 The incorporation of additional literature references to support the newly introduced discussions further strengthens the scholarly foundation of the manuscript. Lastly, the thorough revision of the study's limitations demonstrates the authors' commitment to transparency and rigor in their research endeavors.

 Overall, these revisions significantly enhance the scholarly contribution of the manuscript, positioning it as a valuable resource for academics and practitioners interested in the dynamics of teaching and learning in multicultural educational settings.

Is the work clearly and accurately presented and does it cite the current literature?

Yes

If applicable, is the statistical analysis and its interpretation appropriate?

Partly

Are all the source data underlying the results available to ensure full reproducibility?

Yes

Is the study design appropriate and is the work technically sound?

Yes

Are the conclusions drawn adequately supported by the results?

Partly

Are sufficient details of methods and analysis provided to allow replication by others?

Partly

Reviewer Expertise:

e-learning, social research methods,  impact studies, population, health and migration studies, youth and women studies, organizational development and human resource management

I confirm that I have read this submission and believe that I have an appropriate level of expertise to confirm that it is of an acceptable scientific standard.

# Response

**Published:** 2024-02-17

**Authors:** Hugo Horta

**Affiliations:** The University of Hong Kong, Hong Kong

We just want to leave a note, further thanking the attention that the reviewer placed on reviewing the article (both concerning the original and revised version). We would also thank the reviewer for the very good comments provided which lead to the improvement of the quality of the article in several ways.

# Reviewer report: Reviewer response for version 1

**DOI:** 10.5256/f1000research.135371.r193776

**Published:** 2023-09-04

**Authors:** Myla Arcinas

**Affiliations:** 1 Department of Sociology and Behavioral Sciences, De La Salle University, Manila, Metro Manila, Philippines

The study's objectives are clearly outlined and underscore the importance of investigating the academic success of international medical students within Chinese universities. The direct influence of this success on licensing exams and future job prospects adds solid motivation for the research. Moreover, the study aims to address a noted research gap regarding the awareness of teaching-related factors that impact students' academic achievements, which is a relevant and worthwhile endeavor.

 The chosen methodology, involving semi-structured interviews with 36 academics from two medical universities in China, is suitable for capturing the nuances of the research topic. The timeframe of November 2020 to January 2021 allows for comprehensive data collection over a reasonable period. The interviews' audio recording, verbatim transcription, and subsequent thematic analysis highlight the rigor in data handling and analysis, ensuring transparency and accuracy in the findings.

 The thematic analysis results provide valuable insights into teaching factors perceived by academics as influential for student success. The identified themes demonstrate a comprehensive exploration of the issue, encompassing elements such as teaching style, language support, resource management, academic attributes, mentoring, and assessment practices. This breadth enhances the credibility of the study's findings and adds depth to its implications. However, it is essential to note that the themes generated can still be improved and clustered into three to create depth and quality in the discussion section.

**Teaching Methodology/Pedagogy and Content Alignment **

- Encompassing challenges in teaching pedagogy
- Alignment of teaching content with license exams

This cluster focuses on the interrelation between teaching methodology and content alignment with licensure exams. Both elements are integral to effective instruction and student preparation. Teaching pedagogy influences content, and aligning content with licensure exams ensures students are adequately prepared for assessments. The combined cluster highlights the synergy between pedagogical strategies and content relevance.

**Educator Qualities/Attributes and Guidance **

- Addressing teacher attributes
- Supervision and guidance by teachers
- Establishment of teacher-student rapport

This cluster centers around the educators' role in student development. Teacher attributes influence their ability to provide effective guidance, mentorship, and establish rapport with students. By grouping these themes, the study delves into how educators' personal qualities impact their capacity to provide meaningful Supervision, guidance and build strong relationships with students.

**Resource Management and Learning Environment **

- Management of teaching resources
- Classroom discipline management
- Assessment methodologies

This cluster revolves around the practical aspects of creating a conducive learning environment. Management of teaching resources, classroom discipline, and assessment methodologies collectively influence the overall learning experience. Efficient resource management contributes to effective instruction, while classroom discipline and assessment methods shape the learning atmosphere. Grouping these themes underlines the importance of maintaining an organized, supportive learning environment.

 Given the current themes presentation, the conclusions drawn from the findings are pragmatic and actionable, emphasizing the need for continuous professional development and resource provision for academic staff. The recommendation for staff training aligns well with the identified teaching factors and offers practical guidance for institutions seeking to enhance teaching quality. The clarity of these conclusions contributes to the study's relevance and potential impact on teaching practices.

 While the study's strengths are evident, a few aspects could be considered for improvement. A broader geographical scope or the inclusion of more diverse medical universities might have added insights into potential variations. Additionally, exploring potential research limitations or biases, such as the interviewees' backgrounds or the context of the two chosen universities, would have enhanced the study's transparency and thoroughness.

 In summary, the study's objectives are well-established and significant within the academic context. The methodology is appropriate for the research aim, and the results' thematic analysis provides a comprehensive understanding of teaching factors affecting student success. The study's conclusions offer practical recommendations for improving teaching quality. While the study's strengths are evident, considering a broader scope and addressing potential limitations would further enhance its credibility and impact. It is important to note that clustering of the themes generated could definitely  improve the discussion section and the overall quality of the paper.

 Additional Points for Improvement of the Quality of the Paper:

1. In the Abstract, Methods section, it is crucial to explicitly mention the use  of purposive sampling and state specific criteria employed in selecting informants.
2. In the paper, additionally, within the methods section, clarify the criteria used to identify the two medical universities covered  in the study.
3. When describing the study's sources of data, avoid using "participants" unless it pertains to scenarios wherein  focused group discussions or participant observation was used as method of data collection. In employing semi-structured interviews for data collection, the appropriate terminology is "informant."
4. Please exercise caution in providing qualitative interpretation  derived from descriptive statistics presented in the paper. For instance:
  :
  - Instead of "An overwhelming majority (86%)," employs the term "most" as it correctly corresponds to the range of 75% to 90% in relation to descriptive statistics.
  - For the statement "Approximately 50% of interviewees," provide the actual frequency count and rephrase it to "Nearly half (mention the frequency count)."
  - Similarly, for expressions like "nearly 50% of the interviewees," provide the precise frequency count, for instance, "Nearly half or majority (mention the frequency)."
  - When discussing "Approximately 31% of the interviewees," explicitly mention the factual frequency count.
5. When commencing a sentence with a numerical value, spell out the number. For instance, instead of writing "45 universities are authorized by the MOE…," use "Forty-five universities are authorized by the MOE…"
6. The overall quality of the paper can be further improved through thorough proofreading and editing. For example, consider:
*"Nine key themes emerged covering challenges: teaching pedagogy, English language, teaching resources management, teacher attributes, teacher supervision and guidance, teacher-student rapport, detachment of teaching content from license exams, class discipline management, and assessment styles.*"
  It can be written this way -
  Nine prominent themes surfaced, encompassing challenges spanning teaching pedagogy, English language proficiency, management of teaching resources, teacher attributes, Supervision and guidance by teachers, establishment of teacher-student rapport, alignment of teaching content with license exams, classroom discipline management and assessment methodologies.
7. Two themes can be combined into one theme - Theme:  teacher attributes
  Theme: Supervision and guidance by teachers
  Combined Theme: Teacher attributes and Supervision, or Teacher attributes and Guidance
  Combining these two themes into a single category of "Teacher Attributes and Supervision/Guidance" is justified by the intrinsic relationship between these aspects within the context of education. Teacher attributes inherently influence the quality of Supervision and guidance they provide students. Teachers' personal qualities, experience, and expertise significantly impact their effectiveness in guiding and mentoring students. By grouping these themes, the study can more comprehensively explore how specific teacher attributes contribute to the efficacy of their Supervision and guidance, leading to a deeper understanding of educators' role in student success.

Is the work clearly and accurately presented and does it cite the current literature?

Yes

If applicable, is the statistical analysis and its interpretation appropriate?

Partly

Are all the source data underlying the results available to ensure full reproducibility?

Yes

Is the study design appropriate and is the work technically sound?

Yes

Are the conclusions drawn adequately supported by the results?

Partly

Are sufficient details of methods and analysis provided to allow replication by others?

Partly

Reviewer Expertise:

e-learning, social research methods,  impact studies, population, health and migration studies, youth and women studies, organizational development and human resource management

I confirm that I have read this submission and believe that I have an appropriate level of expertise to confirm that it is of an acceptable scientific standard, however I have significant reservations, as outlined above.

# Response

**Published:** 2024-02-01

**Authors:** Hugo Horta

**Affiliations:** The University of Hong Kong, Hong Kong

**Responses to the comments from F1000 paper:**

**Reviewer #2**

**Comment**: 1.The study's objectives are clearly outlined and underscore the importance of investigating the academic success of international medical students within Chinese universities. The direct influence of this success on licensing exams and future job prospects adds solid motivation for the research. Moreover, the study aims to address a noted research gap regarding the awareness of teaching-related factors that impact students' academic achievements, which is a relevant and worthwhile endeavor.

**Response**: *Thank you very much for your encouraging acknowledgement of the significance of our study. We are delighted that the reviewer feels that our work provides a valuable contribution to the advancement of knowledge.*

**Comment**: 2. The chosen methodology, involving semi-structured interviews with 36 academics from two medical universities in China, is suitable for capturing the nuances of the research topic. The timeframe of November 2020 to January 2021 allows for comprehensive data collection over a reasonable period. The interviews' audio recording, verbatim transcription, and subsequent thematic analysis highlight the rigor in data handling and analysis, ensuring transparency and accuracy in the findings.

**Response**: *Many thanks for this positive assessment of our work and our methodological choices, as well as the collection and analysis of the data.*

**Comment**: 3.The thematic analysis results provide valuable insights into teaching factors perceived by academics as influential for student success. The identified themes demonstrate a comprehensive exploration of the issue, encompassing elements such as teaching style, language support, resource management, academic attributes, mentoring, and assessment practices. This breadth enhances the credibility of the study's findings and adds depth to its implications. However, it is essential to note that the themes generated can still be improved and clustered into three to create depth and quality in the discussion section.

**Response**: *We are extremely thankful again, this time for the positive remarks concerning the analysis. We have tried to re-write the analysis thematically into three themes as the reviewer suggested but ended up reverting some of the changes and decided to have four themes, which are independent even if somewhat overlapping with one another. This – in our opinion – makes the text to flow better and for the readers to have a clearer understanding of the different facets of the analysis. In this regard, we have structured better the sub-themes of the themes presented, many thanks!*

**Comment**: 4. Teaching Methodology/Pedagogy and Content Alignment 

- Encompassing challenges in teaching pedagogy 
- Alignment of teaching content with license exams

This cluster focuses on the interrelation between teaching methodology and content alignment with licensure exams. Both elements are integral to effective instruction and student preparation. Teaching pedagogy influences content, and aligning content with licensure exams ensures students are adequately prepared for assessments. The combined cluster highlights the synergy between pedagogical strategies and content relevance.

 5. Educator Qualities/Attributes and Guidance 

- Addressing teacher attributes 
- Supervision and guidance by teachers 
- Establishment of teacher-student rapport

This cluster centers around the educators' role in student development. Teacher attributes influence their ability to provide effective guidance, mentorship, and establish rapport with students. By grouping these themes, the study delves into how educators' personal qualities impact their capacity to provide meaningful Supervision, guidance and build strong relationships with students.

**6. Resource Management and Learning Environment **

- Management of teaching resources 
- Classroom discipline management 
- Assessment methodologies

This cluster revolves around the practical aspects of creating a conducive learning environment. Management of teaching resources, classroom discipline, and assessment methodologies collectively influence the overall learning experience. Efficient resource management contributes to effective instruction, while classroom discipline and assessment methods shape the learning atmosphere. Grouping these themes underlines the importance of maintaining an organized, supportive learning environment.

 Given the current themes presentation, the conclusions drawn from the findings are pragmatic and actionable, emphasizing the need for continuous professional development and resource provision for academic staff. The recommendation for staff training aligns well with the identified teaching factors and offers practical guidance for institutions seeking to enhance teaching quality. The clarity of these conclusions contributes to the study's relevance and potential impact on teaching practices.

**Response**: *Thank you very much for the reviewer’s positive view on the relevance and content of themes 4, 5 and 6.*

**Comment**: 7. While the study's strengths are evident, a few aspects could be considered for improvement. A broader geographical scope or the inclusion of more diverse medical universities might have added insights into potential variations. Additionally, exploring potential research limitations or biases, such as the interviewees' backgrounds or the context of the two chosen universities, would have enhanced the study's transparency and thoroughness.

**Response**: *We agree that the inclusion of more diverse medical universities such as from different provinces in China would have enhanced the rigor in data and added insights into potential variations. This is one of the limitations of our study that we addressed in the paper. Practically it was a bit difficult to involve more universities especially from other provinces, but we aim to try to make this happen in our future studies.*

*According to the comment of the other reviewer, we provided a table with the informants’ demographic information and their backgrounds. Meanwhile, potential research limitations are now addressed in the Conclusion section.*

**Comment**: 8. In summary, the study's objectives are well-established and significant within the academic context. The methodology is appropriate for the research aim, and the results' thematic analysis provides a comprehensive understanding of teaching factors affecting student success. The study's conclusions offer practical recommendations for improving teaching quality. While the study's strengths are evident, considering a broader scope and addressing potential limitations would further enhance its credibility and impact. It is important to note that clustering of the themes generated could definitely improve the discussion section and the overall quality of the paper.

**Response**: *There are several limitations of the study. This study was conducted at two sites, but caution with generalization of the findings is needed. They may be relevant to other Chinese universities offering courses to international students, but not to other settings. Both research sites were located within one single province, although representative of the general MBBS education in other universities in the same province, a broader geographical scope or the inclusion of more diverse medical universities from other regions or provinces might have added insights into potential variations. Additionally, as the data was sourced from subjective opinions of the academics, potential biases may exist.*

*We have regrouped the themes according to your insightful suggestions in the discussion of the findings.*

  

1. **Comment**: Additional Points for Improvement of the Quality of the Paper: 1. In the Abstract, Methods section, it is crucial to explicitly mention the use of purposive sampling and state specific criteria employed in selecting informants.

**Response**: *Thank you for this helpful comment. We have now added the use of purposive sampling and specific criteria employed in selecting our informants in the Abstract.*

**Comment**: Additional Points for Improvement of the Quality of the Paper: 2. In the paper, additionally, within the methods section, clarify the criteria used to identify the two medical universities covered in the study.

**Response**: *Many thanks. The criteria used to identify the two medical universities have been clarified in the Methods section.*

**Comment**: Additional Points for Improvement of the Quality of the Paper: 3. When describing the study's sources of data, avoid using "participants" unless it pertains to scenarios wherein focused group discussions or participant observation was used as method of data collection. In employing semi-structured interviews for data collection, the appropriate terminology is "informant."

**Response**: *Thank you for this important point. All the “participants” have been replaced with the terminology of “informants” now in the paper.*

**Comment**: Additional Points for Improvement of the Quality of the Paper: 4. Please exercise caution in providing qualitative interpretation derived from descriptive statistics presented in the paper. For instance:

- Instead of "An overwhelming majority (86%)," employs the term "most" as it correctly corresponds to the range of 75% to 90% in relation to descriptive statistics.
- For the statement "Approximately 50% of interviewees," provide the actual frequency count and rephrase it to "Nearly half (mention the frequency count)."
- Similarly, for expressions like "nearly 50% of the interviewees," provide the precise frequency count, for instance, "Nearly half or majority (mention the frequency)."
- When discussing "Approximately 31% of the interviewees," explicitly mention the factual frequency count.

**Response**: *We appreciate this comment and decided to adopt it in the paper. Changes have been made in relevant places.*

**Comment**: Additional Points for Improvement of the Quality of the Paper: 5. When commencing a sentence with a numerical value, spell out the number. For instance, instead of writing "45 universities are authorized by the MOE…," use "Forty-five universities are authorized by the MOE…"

**Response**: *Corrected. Thank you.*

**Comment**: Additional Points for Improvement of the Quality of the Paper: 6. The overall quality of the paper can be further improved through thorough proofreading and editing. For example, consider:

*"Nine key themes emerged covering challenges: teaching pedagogy, English language, teaching resources management, teacher attributes, teacher supervision and guidance, teacher-student rapport, detachment of teaching content from license exams, class discipline management, and assessment styles.*"

 It can be written this way - Nine prominent themes surfaced, encompassing challenges spanning teaching pedagogy, English language proficiency, management of teaching resources, teacher attributes, Supervision and guidance by teachers, establishment of teacher-student rapport, alignment of teaching content with license exams, classroom discipline management and assessment methodologies.

**Response**: *Thank you very much for this point and for your good example of rewriting this paragraph. We agree that some proofreading and editing can improve the quality of the paper. Therefore, we have sent the paper for thorough proofreading and editing with an online academic proofreading service.*

**Comment**: Additional Points for Improvement of the Quality of the Paper: 7. Two themes can be combined into one theme - Theme:  teacher attributes

 Theme: Supervision and guidance by teachers Combined Theme: Teacher attributes and Supervision, or Teacher attributes and Guidance - Combining these two themes into a single category of "Teacher Attributes and Supervision/Guidance" is justified by the intrinsic relationship between these aspects within the context of education. Teacher attributes inherently influence the quality of Supervision and guidance they provide students. Teachers' personal qualities, experience, and expertise significantly impact their effectiveness in guiding and mentoring students. By grouping these themes, the study can more comprehensively explore how specific teacher attributes contribute to the efficacy of their Supervision and guidance, leading to a deeper understanding of educators' role in student success

**Response**: *There is now a teacher attributes and guidance theme, which includes the sub-themes “addressing teaching attributes”, “supervision and guidance by teachers”, and “establishment of teacher-student rapport” . Many thanks for the fantastic suggestion.*

# Reviewer report: Reviewer response for version 1

**DOI:** 10.5256/f1000research.135371.r193769

**Published:** 2023-09-04

**Authors:** Muhammad Azeem Ashraf

**Affiliations:** 1 Research Institute of Education Science, Hunan University, Changsha, Hunan, China

The article is focused on an important topic, and limited data is available on this topic, especially in English. The paper is written well and applies appropriate methods to collect and analyze the data. Authors may consider a few suggestions to improve the article. 

 1. Introduction provides good information about the context, quality, structure, and previous literature on medical education in China. The third paragraph is better merged with the other paragraph, as a one sentence paragraph looks strange. It would add more value to the section if some background information were provided on medical teachers training in other countries. The recent two decades have seen a massive flow of Chinese medical professionals to go abroad for higher studies. And these medical professionals with foreign qualifications take a central position in international medical education. 

 2. Methods: It is written well and provides sufficient information on methods applied in the research. A table on participants' background information is necessary. It will help readers get the participants' essential information while reading their reflections in the results section. Did any participants have foreign qualifications or working or learning experience in a foreign country? 

 3. It would be interesting if the comparison is provided between teachers who have had foreign learning/working experience and teachers who have never been to foreign learning/working. Did teachers reflect on the challenges of diverse classrooms (with students from different backgrounds/nationalities/religions) and students' English proficiency? 

 4. Practical and theoretical implications are better reported in the article, as the study is important to policy-making institutions to improve the teaching and learning of medical education in universities.

Is the work clearly and accurately presented and does it cite the current literature?

Yes

If applicable, is the statistical analysis and its interpretation appropriate?

Not applicable

Are all the source data underlying the results available to ensure full reproducibility?

Yes

Is the study design appropriate and is the work technically sound?

Yes

Are the conclusions drawn adequately supported by the results?

Yes

Are sufficient details of methods and analysis provided to allow replication by others?

Partly

Reviewer Expertise:

Medical education

I confirm that I have read this submission and believe that I have an appropriate level of expertise to confirm that it is of an acceptable scientific standard, however I have significant reservations, as outlined above.

# Response

**Published:** 2024-02-01

**Authors:** Hugo Horta

**Affiliations:** The University of Hong Kong, Hong Kong

**Responses to the comments from F1000 paper:**

**Reviewer #1**

**Comment**: 1. Introduction provides good information about the context, quality, structure, and previous literature on medical education in China. The third paragraph is better merged with the other paragraph, as a one sentence paragraph looks strange. It would add more value to the section if some background information were provided on medical teachers training in other countries. The recent two decades have seen a massive flow of Chinese medical professionals to go abroad for higher studies. And these medical professionals with foreign qualifications take a central position in international medical education. 

**Response**: *We are extremely thankful for this comment. The location of the third paragraph was misplaced by mistake. Now it has been placed where it should be and the text flows much better. Many thanks.*

*We are highly appreciative of this insightful comment regarding the training of medical teachers. Recent and relevant literature on this topic has been added in a new paragraph in the Introduction to address this issue.*

**Comment**: 2. Methods: It is written well and provides sufficient information on methods applied in the research. A table on participants' background information is necessary. It will help readers get the participants' essential information while reading their reflections in the results section. Did any participants have foreign qualifications or working or learning experience in a foreign country? 

**Response**: *Thanks for this good suggestion. We have added a table with the participants’ demographic information. As the reviewer can observe from the table, most of the participants had education, working or training experience in a foreign country, predominantly in the USA.*

**Comment**: 3. It would be interesting if the comparison is provided between teachers who have had foreign learning/working experience and teachers who have never been to foreign learning/working. Did teachers reflect on the challenges of diverse classrooms (with students from different backgrounds/nationalities/religions) and students' English proficiency? 

**Response**: *This is a very good suggestion. We carefully reviewed our data again and found out that there were no major differences between teachers who had overseas learning or working experience and those who had not on the challenges they faced. The teachers in our study seemed to face overall very similar challenges and barriers in terms of diverse classrooms and international students’ English proficiency.*

**Comment**: 4. Practical and theoretical implications are better reported in the article, as the study is important to policy-making institutions to improve the teaching and learning of medical education in universities.

**Response**: *Thank you very much.*
